# Brain and cancer associated binding domain mutations provide insight into CTCF’s relationship with chromatin and its ability to act as a chromatin organizer

**DOI:** 10.21203/rs.3.rs-4670379/v1

**Published:** 2024-07-19

**Authors:** Catherine Do, Guimei Jiang, Giulia Cova, Christos C. Katsifis, Domenic N. Narducci, Jie Yang, Theodore Sakellaropoulos, Raphael Vidal, Priscillia Lhoumaud, Aristotelis Tsirigos, Faye Fara D. Regis, Nata Kakabadze, Elphege P Nora, Marcus Noyes, Xiaodong Cheng, Anders S. Hansen, Jane A Skok

**Affiliations:** 1. Department of Pathology, NYU Grossman School of Medicine, New York, NY, USA; 2. Perlmutter Cancer Center, NYU Langone Health, New York, NY, USA; 3. MIT Department of Biological Engineering; 4. Gene Regulation Observatory, Broad Institute of MIT and Harvard, Cambridge, MA 02139, USA; 5. Koch Institute for Integrative Cancer Research, Cambridge, MA, 02139, USA; 6. Department of Epigenetics and Molecular Carcinogenesis, The University of Texas MD Anderson Cancer Center, Houston, TX 77030; 7. Applied Bioinformatics Laboratories, Office of Science & Research, NYU Grossman School of Medicine, New York, NY, USA; 8. Cardiovascular Research Institute, and Department of Biochemistry and Biophysics, University of California San Francisco, CA, USA; 9. Institute for Systems Genetics, Department of Biochemistry and Molecular Pharmacology, NYU Langone Health, New York, NY, USA

## Abstract

Although only a fraction of CTCF motifs are bound in any cell type, and approximately half of the occupied sites overlap cohesin, the mechanisms underlying cell-type specific attachment and ability to function as a chromatin organizer remain unknown. To investigate the relationship between CTCF and chromatin we applied a combination of imaging, structural and molecular approaches, using a series of brain and cancer associated CTCF mutations that act as CTCF perturbations. We demonstrate that binding and the functional impact of WT and mutant CTCF depend not only on the unique properties of each protein, but also on the genomic context of bound sites. Our studies also highlight the reciprocal relationship between CTCF and chromatin, demonstrating that the unique binding properties of WT and mutant proteins have a distinct impact on accessibility, TF binding, cohesin overlap, chromatin interactivity and gene expression programs, providing insight into their cancer and brain related effects.

## Introduction

The CCCTC-binding factor (CTCF) is an eleven zinc finger DNA-binding protein that plays a key role in chromatin organization and gene regulation. Key insight into the mechanisms underlying CTCF’s function were revealed by molecular studies showing that cohesin binding overlaps CTCF sites on chromatin in a CTCF dependent manner ^1,2^. A more coherent picture of the co-operative function of these two factors emerged from subsequent analyses showing that acute depletion of CTCF in cell lines leads to loss of highly self-interacting topologically associated domain (TAD) structures and redistribution of cohesin on chromatin ^4^. It is now well established that CTCF and cohesin play a key role in organizing chromatin into TAD structures by promoting the formation of loops and boundaries that are important for gene regulation. This involves a loop-extrusion mechanism in which cohesin complexes create loops by actively extruding DNA until movement of the complex is blocked by two CTCF binding sites in convergent orientation ^5,6^. The requirement for orientation specific CTCF binding can be explained by the underlying structure of the interaction between the two proteins, which occurs between the SA2-SCC1 component of cohesin and the N terminal region of CTCF ^7–10^. CTCF, in conjunction with cohesin, is enriched at TAD boundaries that function as insulators, contributing to gene regulation by restricting the interaction of regulatory elements to promoters of target genes located within the same TAD.

While the above studies provide critical insight into CTCF’s global contribution to chromatin folding and gene regulation, the role of CTCF in regulating individual loci in a context specific manner is unclear. Since CTCF’s binding profiles are known to be cell-type specific chromatin accessibility is presumed to be important for CTCF attachment. However, the mechanisms underlying CTCF’s ability to bind and act as a chromatin organizer in a cell-type specific manner are incompletely elucidated. CTCF has been degraded by auxin in numerous cell types, and although these studies provide important insight into its role in gene regulation, these models are insufficient for (i) analyzing the contribution of genomic context to site-specific CTCF binding and function, or (ii) distinguishing direct from indirect effects that can be a confounding issue for interpreting site specific impact. Instead, information related to CTCF’s direct effects on individual loci has come from the genetic ablation of CTCF binding sites at specific regions in the genome in a given cell type. Indeed, disruption of TAD boundaries by deletion of one or more proximal binding sites can alter gene regulation as a result of aberrant enhancer promoter contacts, and this can have dramatic consequences on developmental processes and cancer initiation ^11–16^. However, deletion of CTCF boundary elements can be contextual, impacting gene expression in certain cell types but not in others. Furthermore, interrogating the general mechanisms underlying the crosstalk between CTCF function and the cell- and locus-specific context of its binding by genetic manipulation of individual CTCF binding sites is laborious and has the limitation of providing insight into the control of only a limited number of loci in the neighboring region.

The *CTCF* locus, located on chromosome 16q band 22, corresponds to one of the smallest regions of overlap for common deletions in breast and prostate cancers ^16,17^. Moreover, point mutations and deletions have been identified in many other tumors. Together these findings indicate that CTCF acts as a tumor suppressor ^18^. Consistent with this, deletion of one *Ctcf* allele predisposes mice to spontaneous B-cell lymphomas as well as radiation- and chemically-induced cancer in a broad range of tissues ^19^. Additionally, genetic alterations in *Ctcf* and changes in dosage are associated to varying degrees with intellectual disability and microcephaly, and thus CTCF is thought to play an important role in brain development and neurological disorders ^20–22^.

CTCF binds to chromatin through a subset of its eleven zinc fingers (ZFs) ^23,24^. Specifically, ZFs3–7 make sequence specific contacts with a 12–15 base-pair (bp) core consensus binding site and it is thought that ZF2, ZF8 and ZF9 contribute to stability (Hashimoto, Wang et al. 2017). ZF1 and ZF10, contain RNA binding domains (RBDs) that contribute to binding of CTCF and CTCF-mediated looping at a subset of sites through an unknown mechanism ^25^. While the crystal structure of CTCF in complex with a known DNA binding domain has revealed new insights into the contribution of each ZF to sequence-specific binding in an *in vitro* setting ^26^, there has been no complementary, comprehensive analysis of cancer associated CTCF mutants and it is thus not known how mutations within each ZF impact binding stability and DNA sequence specificity, and the consequences this has on binding profile, cohesin overlap, accessibility, chromosome architecture and gene regulation. Given this lack of information, we have no context in which to determine the functional relevance of mutations.

Using an inducible mouse ESC complementation system, we combined imaging, structural, molecular and bioinformatic analyses to examine the impact of nine, high frequency cancer associated CTCF mutations in eight amino acid residues of ZFs that make contact with the core consensus binding motif. A subset of these are also associated with neurological disorders. Collectively, the nine mutations have a graded impact and can act as CTCF perturbations, providing an elegant system with which to investigate, (i) the relationship between WT and mutant CTCF and chromatin, and (ii) site specific mutant effects on accessibility, TF binding, cohesin overlap, chromatin interactivity and gene expression, both of which offer insight into CTCF’s biology and its cancer and brain related effects. Compared to a complete knockout of CTCF or deletion of a CTCF binding site, the mutants offer a more subtle and in-depth approach for teasing out the contribution of CTCF to chromatin organization and gene regulation, since each mutation produces its own unique effect.

Here we demonstrate that the functional impact of WT and mutant CTCF depends not only on the structural effects of each mutation on the protein’s binding properties, but also on the genomic context of the binding sites. Analyses of the relationship between CTCF, cohesin and accessibility revealed that although CTCF can bind both accessible and inaccessible sites, it has a weaker signal and is less competent at performing its function of blocking cohesin at inaccessible sites. CTCFs ability to block cohesin is also linked to each mutants residence time or ‘OFF’ rate on chromatin as determined by FRAP experiments, while occupancy or ‘ON’ rate is correlated with their binding at common accessible sites, indicating that accessibility dominates the search space. Together, the mutants highlight the fact that occupancy and residence time can be uncoupled such that although some mutants have fewer binding sites than WT CTCF, they have a stronger residence time at these locations which increases their ability to block cohesin and contribute to chromatin folding. The reverse situation also occurs such that mutants which occupy more sites than WT, do so in an unstable manner with low residence times that impair their ability to engage cohesin and form loops.

Interestingly, although accessibility can impact CTCF binding, CTCF can in turn reduce accessibility at locations where it can block cohesin, suggesting a role for loop extrusion in altering accessibility. The mutations enable us to determine that CTCF binding drives the reduction in accessibility, rather than the other way around because each mutant has a distinct ability to alter accessibility depending on its binding stability. The unique properties of WT and mutant CTCF also effect TF binding and cancer, brain, immune and metabolic gene expression pathways. Unexpectedly, mutants have a more pronounced effect on transcriptional output than auxin inducible degradation, likely due to indirect effects that result from altered TF binding profiles. Collectively, the mutants offer a rich resource to investigate site specific CTCF-mediated effects on chromatin folding and gene regulation, while distinguishing cause from consequence and direct from indirect outcomes. Finally, graded mutant perturbations provide mechanistic insight into CTCF biology and mutant specific cancer and brain related effects.

## RESULTS

### CTCF complementation system

Although it is possible to examine CTCF mutations in cancer cells to determine their functional outcome, this setting is not ideal because the latter have many other genetic and epigenetic alterations that would confound a clean analysis of their impact. To circumvent these issues, we made use of a small-molecule auxin inducible degron (AID) mouse ESC (mESC) system ^4,8^. In these cells, both endogenous CTCF alleles are tagged with AID as well as eGFP (CTCF-AID-eGFP) and the auxin-activated ubiquitin ligase TIR1 (from *Oryza sativa*) is constitutively expressed from the *Rosa26* locus*.* Addition of indole acetic acid (IAA, an analogue of auxin) leads to rapid poly-ubiquitination and proteasomal degradation of the proteins tagged with the AID domain.

**Fig. 1:**
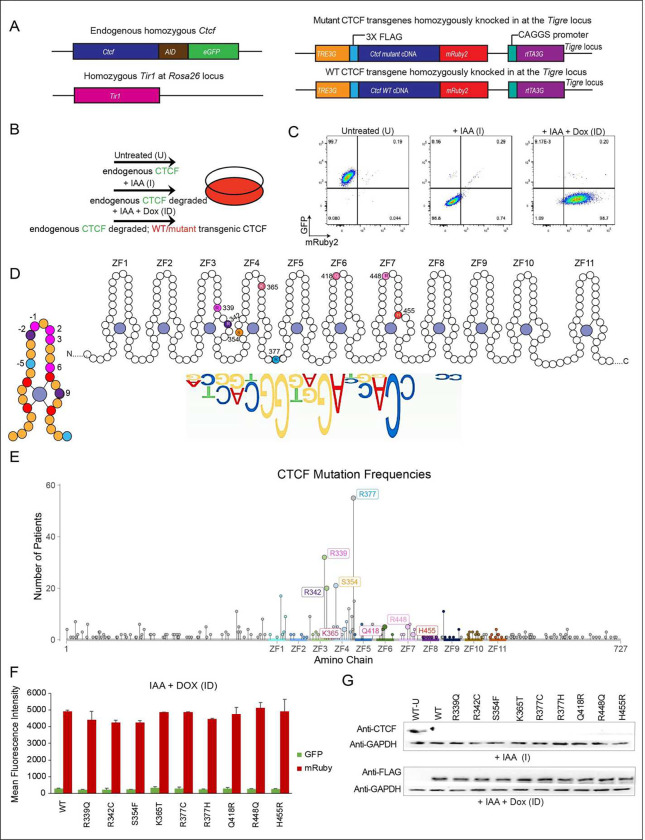
CTCF complementation system. (**A)** Scheme of genetic modifications in the *Ctcf* locus and the doxycycline inducible knocked-in WT and mutant transgenic *Ctcf* at the *Tigre* locus. The endogenous *Ctcf* contains the auxin inducible degron (AID) and the eGFP tag on both alleles. All transgenes harbor an N terminal 3 X FLAG tag and *TetO-3G* element as well as a C terminal mRUBY2 and *rtTA3G* for doxycycline induced expression. (**B**) Experimental strategy for expression of dox-inducible WT and mutant transgenic CTCF in the absence of endogenous CTCF using the auxin inducible degron system. Addition of indole-3-acetic acid (IAA) a chemical analog of auxin leads to transient and reversible degradation of endogenous CTCF, while addition of doxycycline (Dox) leads to induction of transgene expression. The three conditions used in our analysis are: U, untreated cells; I, IAA treated for CTCF depletion; ID, IAA plus Dox treated for depletion of endogenous CTCF and induction of WT or mutant transgenic CTCF expression. (**C**) Flow cytometry showing the level of GFP (endogenous CTCF) and (transgenic WT CTCF) under the different conditions shown in (**B**). (**D**) Scheme showing the locations of the different types of CTCF mutations within a ZF (left). Amino acids that make base-specifying contacts with the alpha helix of DNA (at locations −2, 2, 3 and 6 on the ZF are shown in different shades of pink, residues that coordinate the zinc ion are shown in red, boundary residues are shown in purple, and residues that contact the sugar phosphate backbone of DNA are shown in blue. Schematic representation of CTCF showing the locations of each mutation under investigation. (**E**) Graph showing the incidence and location of CTCF mutations in cancer patients. The color codes match those in **D**. (**F**) Bar graph showing the expression levels of transgenic (mean fluorescence intensity), mRuby2 labelled WT and mutant CTCF after treatment with IAA and Dox (ID). The presence of the IAA leads to degradation of GFP labelled endogenous CTCF. The error bars represent the standard deviation between the two replicates. (**G**) Western blot showing degradation of endogenous CTCF as detected with an antibody to CTCF, and the induction of transgenic CTCF as detected using an antibody to FLAG. The second replicate is shown in Extended Data Fig. 2.

To study the impact of CTCF mutations, we established a rescue system wherein the mESC degron cell line was modified to express either a stable doxycycline-inducible control wild-type *Ctcf* or a mutant *Ctcf* (*mCtcf*) transgene in the absence of endogenous CTCF (Nora, Caccianini et al. 2020) (Fig. 1A). Transgenes have a 3 x FLAG tag, which allows us to distinguish mutant or wild-type transgenic CTCF from endogenous CTCF using a FLAG antibody in Western blot and ChIP-seq analysis. An mRuby fluorescent tag enables us to accurately determine expression levels of mutant or wild-type transgenic CTCF by FACS. The three conditions used for our analysis are shown in Fig. 1B, and an example of the FACS profile for cells expressing a WT CTCF transgene in each condition is shown in Fig. 1C. This system was previously used by us to study the interplay between CTCF and CTCFL, the paralog of CTCF ^8^.

**Extended Data Fig. 1:**
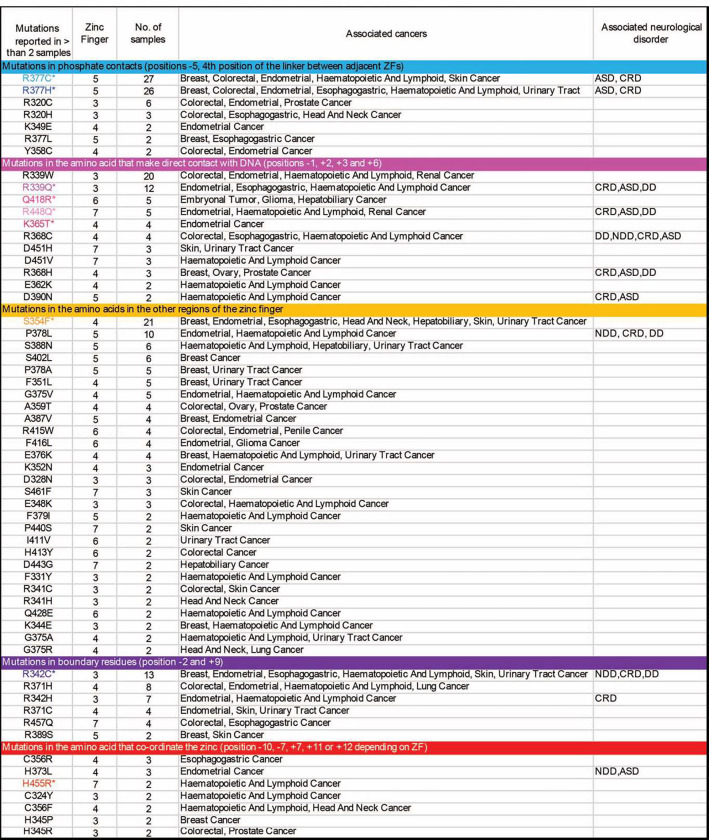
Table of CTCF mutations. **Table showing CTCF** mutations identified from cBioPortal^27,29^ and COSMIC (http://www.sanger.ac.uk/genetics/CGP/cosmic/). We selected a subset of mutations that occur at high frequency in cancer patients, focusing on those found in ZFs that make contact with the 12–15bp consensus CTCF binding motif. These are highlighted with an asterisk and color coded as in Fig. 1D. Mutations analyzed included those found in **(i)** amino acids that make base-specifying contacts with the alpha helix of DNA (at locations −2, 2, 3 and 6 on the ZF), **(ii)** residues that coordinate the zinc ion which are essential for providing stability to ensure the proper folding of the ZF domain, **(iii)** two other classes of amino acid residues in the ZFs: boundary residues (that are important for interactions between ZFs), and residues that contact the sugar phosphate backbone of DNA, and **(iv)** other highly mutated residues in the region. The table identifies which zinc finger each mutation is found in, the number of patients it was identified in and the cancers each is associated with. Mutations were annotated for neurodevelopmental or neurological disorders using the CTCF variant catalog published in Price et al. ^3^. ASD, autism spectrum disorder; CRD, CTCF related neurodevelopmental disorder; DD developmental disorder; NDD, neurodevelopmental disorder.

We selected a subset of missense mutations that occur at high frequency in cancer patients (Extended Data Fig. 1), focusing on those found in ZFs that make contact with the 12–15bp consensus CTCF binding motif (Fig. 1D and highlighted with an asterisk in Extended Data Fig. 1). We analyzed mutations in **(i)** amino acids that make base-specifying contacts with the alpha helix of DNA (at locations −2, 2, 3 and 6 on the ZF), **(ii)** residues that coordinate the zinc ion which are essential for providing stability to ensure the proper folding of the ZF domain, **(iii)** two other classes of amino acid residues in the ZFs: boundary residues (that are important for interactions between ZFs), and residues that contact the sugar phosphate backbone of DNA, and **(iv)** other highly mutated residues in the region. Each class of mutation is colored coded as shown in Fig. 1D (left) and Extended Data Fig. 1, and the color code is maintained throughout the figures. Mutations were identified from cBioPortal ^27–29^ and COSMIC ^30^ (http://www.sanger.ac.uk/genetics/CGP/cosmic/)

**Extended Data Fig. 2:**
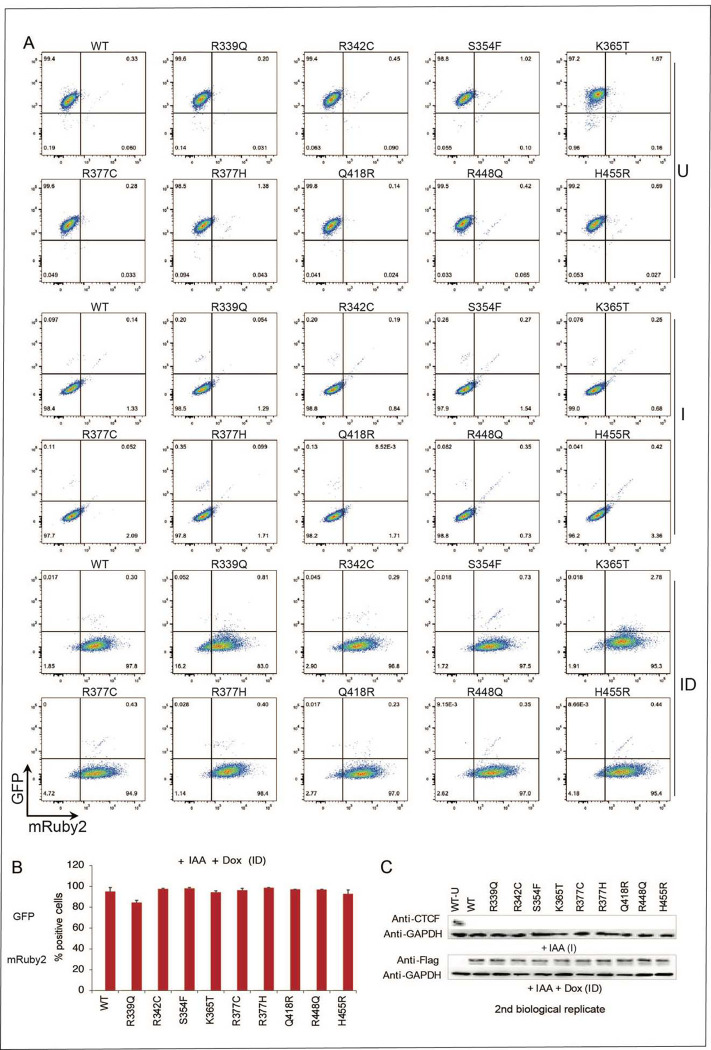
FACS analysis of mutant expression. (A) Flow cytometry showing the level of GFP (endogenous CTCF) in untreated and IAA treated cells. Flow cytometry showing the level of GFP (endogenous CTCF) and mRuby (transgenic CTCF) in the IAA and Dox treated cells (ID). (B) Bar graph showing the percentage of GFP (endogenous CTCF) and mRuby2 (transgene WT and mutant CTCF) positive cells after treatment with IAA and Dox (ID). The presence of the IAA leads to degradation of GFP labelled endogenous CTCF. The error bars represent the standard deviation between the 2 replicates. (**C**) Western blot showing degradation of endogenous CTCF as detected with an antibody to CTCF, and the induction of transgenic CTCF as detected using an antibody to FLAG. The second replicate is shown in Fig. 1.

In total, we analyzed mutations in eight amino acid residues and for R377, the most highly mutated residue in cancer, we included two distinct mutations of R377, the most highly mutated residue in cancer, both of which (R377H and R377C) are equally represented in patients (Fig. 1E and Extended Data Fig. 1). Selected mutations (R339Q, R342C, R448Q, R377H and R377C) have also been shown to be implicated in neurodevelopmental disorders (Extended Data Fig. 1) ^3,22^. To analyze the impact of each CTCF mutation, individual clones with comparable levels of transgene expression (mutant and WT) were selected based on FACS and Western blot analysis (Fig. 1F, G and Extended Data Fig. 2).

### Mutations reduce the chromatin residence time and bound fraction of CTCF

**Fig. 2:**
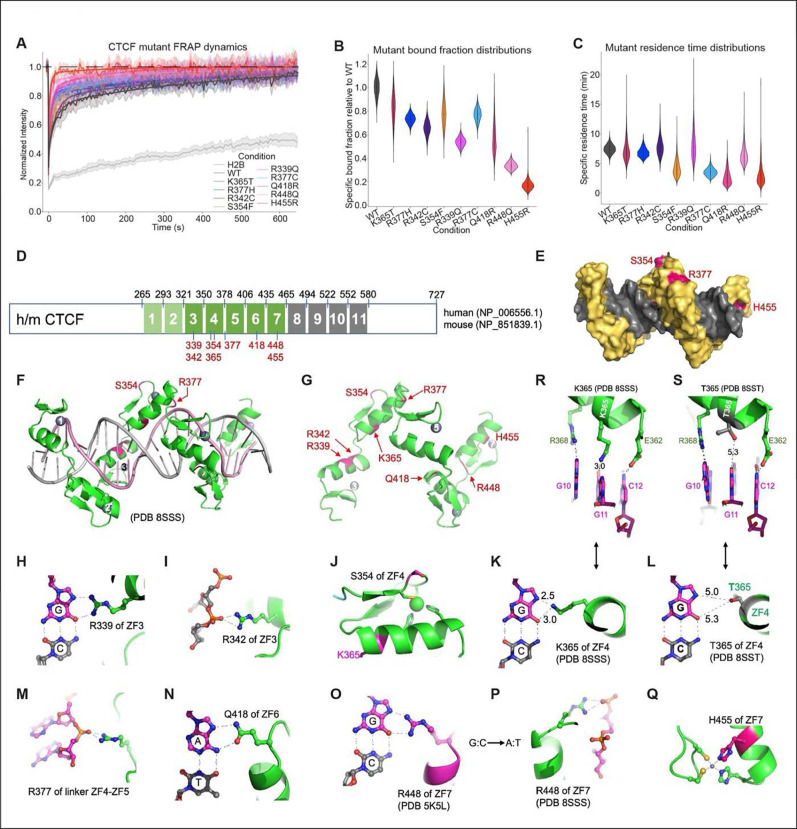
Each mutation uniquely impacts CTCF’s chromatin bound fraction, residence time and interaction with DNA (**A)** Plots of FRAP dynamics for WT and mutant mRuby-CTCF overlaid with a two-state binding model. The scatterplots show the average recovery across all movies analyzed, and the outlines give 95% CI. The bold line shows the fitted model. (**B**) Violin plots of specific bound fraction distributions. (**C**) Violin plots of specific residence time (min) distributions. Specific bound fraction and specific residence time distributions were determined by bootstrapping (n=2500). (**D)** The schematic 11 tandem zinc fingers (ZF) of human (or mouse) CTCF proteins. The amino acid numbers in black indicate the start and end residue of each ZF. The numbers in magenta indicate the mutants observed in ZF3-ZF7 examined in this study. (**E**) Space filling model of ZF1-ZF7 bound with DNA. Three mutated residues (Ser354, Arg377 and His455) are visible from the surface, whereas the others are buried in the protein-DNA interface. **(F)** Ribbon model of ZF1-ZF7 follows the right-handed twist of the DNA, with each finger occupying the DNA major groove (PDB 8SSS). (**G**) The 8 mutants are mapped onto ZF3-ZF7. For clarity, the bound DNA molecule and ZFs outside of ZF3-ZF7 are removed. (**H**) Arg339 of ZF3 recognizes a G:C base pair. (**I**) Arg342 interacts with a DNA backbone phosphate group. (**J**) Ser354 of ZF4 is located between two zinc-coordination cysteine residues and is exposed on the surface away from DNA binding. (**K**) Lys365 of ZF4 interacts with a G:C base pair by forming two hydrogen bonds with the O6 and N7 atoms of guanine. (**L**) The substitution of Lys365 with threonine (K365T) results in loss of direct interactions with the corresponding base pair (PDB 8SST). (**M**) Arg377, located in the linker between ZF4 and ZF5 interacts with a DNA backbone phosphate group. (**N**) Gln418 of ZF6 recognizes an A:T base pair. (**O**) Arg448 of ZF7 recognizes a G:C base pair (PDB 5K5L), and (**P**) when the G:C base pair becomes A:T, Arg448 undergoes a conformational change to interact with a DNA backbone phosphate group (PDB 8SSS). (**Q**) His455 of ZF7 is one of the four Zn-coordination residues. (**R**) Lys365-containing ZF4 recognizes a triplet of three base pairs (GGC). (**S**) Thr365-containing ZF4 interacts with the same GGC triplet without a direct contact with the central guanine.

To elucidate the impact of the cancer-associated mutations on CTCF’s binding dynamics, we performed fluorescence recovery after photobleaching (FRAP) on the 9 mutants and wild-type CTCF ^31,32^. Briefly, we degraded endogenous CTCF-mAID-GFP by IAA addition, induced WT or mutant transgene expression with Dox for 48 hours, and then performed FRAP by bleaching a circular 1 μm diameter circle, and monitored recovery for over 10 minutes, recording 30 movies per condition over three replicates. While the recovery of WT-CTCF largely matched prior CTCF FRAP results ^31^, all mutants exhibit faster recovery consistent with reduced and/or less stable DNA-binding (Fig. 2A). To quantitatively extract dynamic parameters, we fit the FRAP curves to a reaction-dominant kinetic model ^31,32^ and estimated the residence time - duration of CTCF binding to DNA – and bound fraction – proportion of total CTCF bound to DNA – of each mutant with subsequent comparison to the WT (Fig. 2B–C). As shown in Fig. 2B–C, there is a general reduction in the residence time and/or bound fraction across each mutant, with WT CTCF exhibiting the largest specific bound fraction and residence time and the mutants showing a lower or similar residence time. Each mutant displayed unique parameters, regardless of the mutant category. The R377H and R377C mutants – both mutations in the phosphate contacts – have similar bound fractions, but distinct residence times. The mutations in the amino acids making direct contact with DNA – R339Q of ZF3, K365T of ZF4, Q418R of ZF6, and R448Q of ZF7 – have varied residence times and bound fractions, despite belonging to the same group. The R448Q mutant of ZF7 showed the second lowest bound fraction, despite exhibiting a residence time comparable to WT. The Q418R mutant of ZF6 had the lowest residence time of all mutants examined with an intermediate bound fraction. Conversely, the K365T mutant of ZF4 exhibited a bound fraction and residence time most closely comparable to WT. The H455R mutation of ZF7, which assists in coordinating the zinc, had the lowest bound fraction and the second lowest residence time of all examined mutants, accurately reflecting the most deleterious mutation to canonical CTCF function. The R342C mutation of ZF3, which occurs in a boundary residue between the two zinc-ligand histidine residues of ZF3, showed a residence time similar to wild type, but had a lower bound fraction, possibly suggesting a reduced role for boundary residues in binding efficiency. Lastly, the S354F mutant of ZF4, located between the two zinc-ligand cysteine residues of ZF4, is substituted by a bulky phenylalanine in place of a serine in ZF4, showed both a moderate decrease in bound fraction as well as residence time.

In summary, our FRAP results demonstrate that all mutants show changes in DNA binding either through an altered residence time or bound fraction. Surprisingly, some mutations strongly affect either the residence time or the bound fraction, with minimal effects on the other. This suggests that the CTCF target search mechanism (ON-rate) can be decoupled from binding stability (OFF rate) ^33^, such that some mutants affect the efficiency of CTCF search for binding sites, without affecting the residence time of CTCF once bound.

### Mutations uniquely impact CTCF’s interaction with DNA

To complement the molecular profiling of the CTCF mutants, computational modeling of the CTCF protein structure was generated for the eight mutated residues in ZF3-ZF7 under investigation. The potential effects of these missense mutants were examined in the context of available CTCF protein structures, including the core DNA binding domain containing fragments of ZF1-ZF7 (PDB 8SSS), K365T (PDB 8SST), ZF2-ZF7 (PDB 5TOU), ZF3-ZF7 (PDB 5KKQ and 5T00), ZF4-ZF7 (PDB 5K5H), ZF5-ZF8 (PDB 5K5I and 5K5J), ZF6–8 (PDB 5K5L) and ZF4-ZF9 (PDB 5UND) in complex with cognate DNA ^26^.

The location of the eight mutated residues within the CTCF protein is shown in Fig. 2D. These are mapped onto ZF3-ZF7, with the DNA molecule and ZFs outside of ZF3-ZF7 removed for clarity (Fig. 2E). A ribbon model of ZF1-ZF7 following the right-handed twist of the DNA, showing each finger occupying the DNA major groove (PDB 8SSS) is depicted in Fig. 2F. The latter, and a space filling model of ZF1-ZF7 bound to DNA, shows the three mutated residues, Ser354, Arg377 and His455, which are visible from the surface, while the others are buried in the protein-DNA interface (Fig. 2F, G). Figures 2H–S outline the interactions between each of the mutated residues and the DNA.

Four of the eight missense mutants in ZF3-ZF7s are located at DNA-base interacting residues crucial for DNA sequence recognition. These include Arg339 of ZF3, Lys365 of ZF4, Gln418 of ZF6, and Arg448 of ZF7, which are involved in interactions with guanine (via arginine or lysine) and adenine (via glutamine), and thus the substitutions lead to loss of specific DNA interactions and gain of additional binding sites by accommodating other possible base pairs. Lys365-containing ZF4 recognizes a triplet of three base pairs (GGC) and when this is mutated to Thr365, ZF4 interacts with the same GGC triplet without a direct contact with the central guanine, making a less stable interaction. Arg342 of ZF3 and Arg377, located between ZF4 and ZF5, are involved in interactions between the two zinc fingers and DNA backbone phosphate interactions. Thus, changes to these arginine residues might be disruptive for DNA binding stability, and indeed the imaging analyses demonstrate that each mutant has a distinct impact.

Ser354 of ZF4 lies between two zinc coordination cysteine residues, and is positioned on the surface of CTCF, pointing away from DNA binding. S354F/Y introduces a bulky aromatic residue that creates a surface hydrophobic residue that might affect other (non-DNA binding) interactions. Alternatively, S354F/Y could destroy the integrity of ZF4 by affecting zinc binding. However, if the latter were the case one would expect a bigger effect on binding and residence time than that observed (Fig. 2B and C). His455 of ZF7 is one of the (C2H2) residues crucial for coordination of Zn binding to ZF7. The H455R substitution results in loss of zinc ion binding, which would be expected to have a dramatic effect, consistent with the observed loss of binding and residence time shown by our imaging analyses. Together the structural changes inherent to each mutation provide insight into the mutant specific binding properties observed by FRAP.

### CTCF mutations have unique chromatin binding profiles

To supplement the FRAP and structural findings, we next analyzed the binding profiles of mutant versus WT CTCF at the molecular level by performing a FLAG ChIP-seq using cells expressing transgenic WT and mutant CTCF in the absence of endogenous CTCF (ID) using the same auxin and Dox conditions as described for the FRAP experiments. Using this approach we identified three groups of binding sites for each mutation: WT only (sites bound by WT CTCF that mutants can no longer bind), common sites (sites that are bound by both WT and mutant proteins) and mutant only sites (*de novo* sites that only mutant proteins bind).

**Extended Data Fig. 3:**
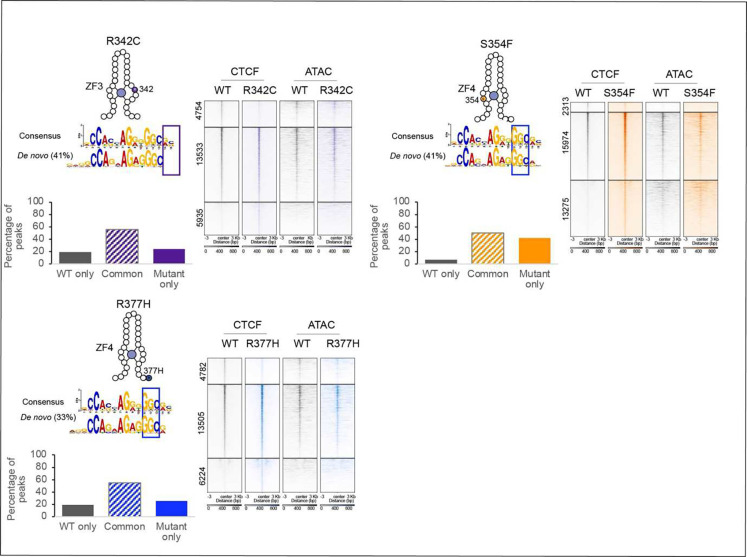
CTCF mutations have unique chromatin binding profiles: Scheme showing the locations of the different CTCF mutations within the ZF. The consensus CTCF motif highlights the triplet to which each mutant zinc finger binds (top) as well as the motif identified by MEME for the *de novo* mutant only binding sites (bottom). Each graph shows the percentage of WT and mutant CTCF binding at WT only, common and mutant only sites and heatmaps show the profile of CTCF binding and ATAC-seq signal for WT only, common and mutant only sites. The remaining mutants are shown in Fig. 3.

**Fig. 3:**
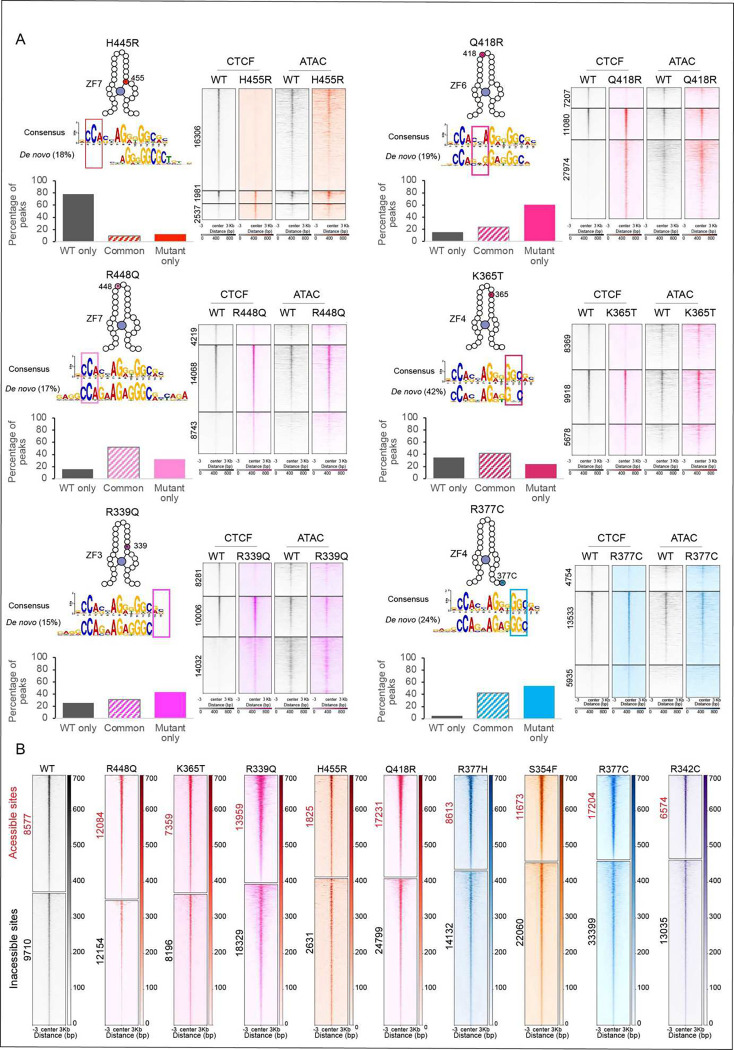
CTCF mutations have unique chromatin binding profiles. (**A)** Scheme showing the locations of the different CTCF mutations within the ZF. The consensus CTCF motif highlights the triplet to which each mutant zinc finger binds (top) as well as the motif identified by MEME for the *de novo* mutant only binding sites (bottom). Each graph shows the percentage of WT and mutant CTCF binding at WT only, common and mutant only sites and heatmaps show the profile of CTCF binding and ATAC-seq signal for WT only, common and mutant only sites. (**B**) Heatmaps show the proportion and number of CTCF binding sites found at accessible and inaccessible chromatin.

The location of each mutation and the complementary DNA triplet that the relevant ZF binds is marked by a box in the consensus motif shown in Fig. 3A and Extended Data Fig. 3A. Below that we highlight the motif identified by MEME ^34^ for the set of *de novo* sites that each mutant binds. These motifs differ from that of the consensus sequence by either (i) a diminished requirement for DNA bases that make contact with the zinc finger that is mutated (Fig. 3A), or (ii) an alteration to the sequence of the consensus motif, which could be explained by the impact of mutations on DNA-base interacting zinc finger residues crucial for DNA sequence recognition. Consistent with the FRAP results, each mutant displays their own unique binding profiles (Fig. 2C) including the two distinct mutations of residue R377, which is located in the linker region between ZF3 and ZF4. R377C binds to less common and more *de novo* sites compared to R377H and each mutant binds a motif that is similar to the consensus motif but lacking bases associated with ZF3. (Fig. 3A and Extended Data Fig. 3A).

The bar graphs show the percentage of the three groups of binding sites for each mutant, while the heatmaps reveal the number of binding sites for each subset and highlight differences in binding strength between WT and mutant at the three locations. It is of note that even at common sites, the strength of CTCF binding is altered in a mutant specific manner, suggesting that a ‘binding dosage effect’ could contribute to their functional impact. As expected, the H455R mutation in the zinc coordinating residue of ZF7 loses the most binding sites and has a high percentage of WT only sites, mirroring the FRAP results (Fig. 2).

To determine whether differences in peak intensity in the different groups could be explained by differences in accessibility, we performed omniATAC ^35^ (Fig. 3A and Extended Data Fig. 3A). Indeed, the profiles for the different binding groups indicate that CTCF binding strength is a function of chromatin accessibility, with common sites, that predominantly have the strongest CTCF signal, being the most accessible. Fig. 3B shows the distribution of binding at inaccessible versus accessible sites for WT and mutant CTCF. The proportion of inaccessible and accessible bound sites are roughly equivalent for WT, R448Q and K365T CTCF, while binding of the remaining mutants is skewed towards inaccessible sites. The heatmaps also underscore the findings from Fig. 3A, showing that accessible binding sites predominantly have stronger CTCF signals compared to inaccessible binding sites.

In sum, ChIP-seq analysis reveals that CTCF mutants lose, retain and gain a subset of binding sites throughout the genome with mutant specific profiles. Loss and gain of CTCF binding occurs predominantly at weak inaccessible CTCF sites, while binding is retained at the stronger common binding sites which have high accessibility, although binding strength and accessibility can vary depending on the mutant. Together with the FRAP and structural data, this analysis suggests that the effect of each mutation can be decoupled into three metrics: number of binding sites, occupancy/bound fraction (ON-rate) and residence time (OFF-rate), which are likely to be the result of a mutant-specific combination of altered sequence-dependent binding and stability as well as differences in chromatin accessibility.

### CTCF mutations have a graded impact on binding and function

**Fig. 4:**
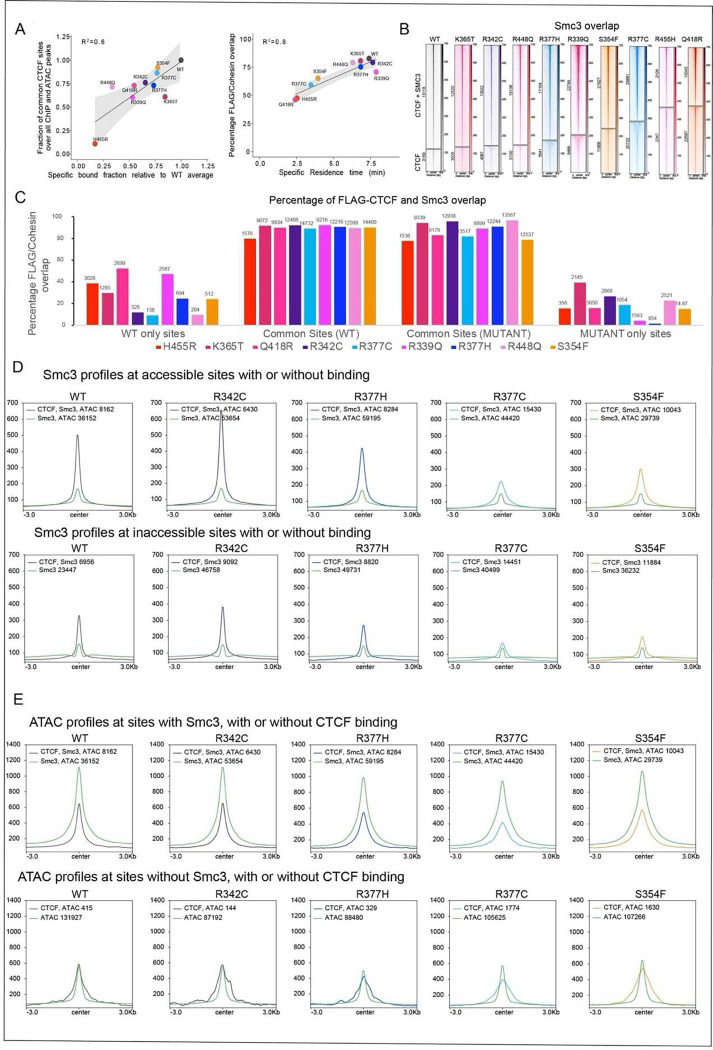
The relationship between CTCF binding, cohesin overlap and accessibility (**A)** Correlation (R^2^=0.6) between the chromatin bound mutant versus WT fraction detected by FRAP and the fraction of mutant binding at common CTCF sites relative to all FLAG ChIP-seq and ATAC-seq peaks (left). Correlation (R^2^=0.8) between residence time and the percentage of CTCF-cohesin overlap for each mutant (right). (**B**) Heatmaps show the proportion and number of CTCF-cohesin versus CTCF only binding sites All CTCF peaks correspond to the peaks found in mutant or WT CTCF expressing cells. (**C)** Bar graph showing the overlap between CTCF and cohesin (Smc3) binding at WT only, common and mutant only sites, in cells expressing either WT or mutant CTCF. (**D)** Profiles of Smc3 binding in WT and mutant CTCF expressing cells is shown for accessible (top) and inaccessible (bottom) sites with (color-coded for each mutant) or without (green) CTCF binding. The remaining mutants are shown in Extended Data Fig. 5A. (**E**) Profiles of ATAC-seq in WT and mutant CTCF expressing cells is shown for accessible Smc3 sites (top) and accessible sites without Smc3, with (color-coded for each mutant) or without (green) CTCF binding. The remaining mutants are shown in Extended Data Fig. 5B. CTCF bound accessible sites have a narrower ATAC-seq peak compared to unbound sites at Smc3 sites. Each CTCF mutant has a differential ability to decrease the breadth of ATAC-seq peaks.

To further understand the relationship between FRAP and ChIP-seq data, we compared the specific bound fraction estimated by FRAP, to the bound fraction estimated by ChIP-seq using FLAG ChIP-seq and ATAC-seq peaks to define all potential binding sites. While we did not find a good correlation when assessing the overall CTCF ChIP-seq bound fraction, we found a strong and significant correlation (R^2^=0.6, p=0.007) between the chromatin bound fraction of the mutants detected by FRAP and the fraction of mutant binding at common CTCF sites (Fig. 4A). These data suggest that the FRAP bound fraction mostly captures the effect of the mutations on the strong accessible binding sites.

We next asked whether the residence time detected by FRAP is linked to CTCF’s binding stability and ability to block cohesin. For this, we performed ChIP-seq for cohesin (using an Ab to the cohesin component, Smc3) and found that the residence time was strongly associated (R^2^=0.8, p=0.0002) with the percentage of CTCF-cohesin overlap for each mutant (Fig. 4A) which reflects the impact of binding stability on CTCF’s function in loop extrusion. As shown in the heatmaps, the proportion of global CTCF-cohesin versus CTCF only binding is graded across mutants, to some extent mirroring a gene dosage effect that occurs as a consequence of altered residence time (Fig. 4B).

**Extended Data Fig. 4:**
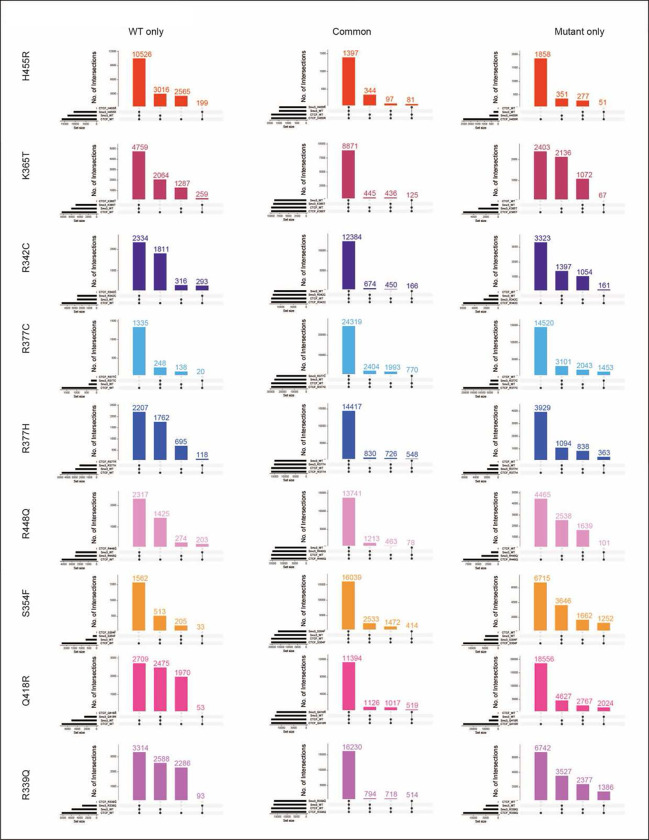
Overlap between CTCF and Cohesin (Smc3) peaks. Upset plots showing for each pairwise comparison between WT and mutant the overlap between WT CTCF, mutant CTCF, WT Smc3 and mutant Smc3 peaks. WT Smc3 sites overlapping with WT only CTCF sites but also present in mutant and conversely, mutant Smc3 sites overlapping with mutant only CTCF sites but also present in WT were considered CTCF-independent for the analysis shown in Fig. 4C.

To examine the effect of mutations on CTCF’s function in loop extrusion, we assessed the overlap of WT and mutant CTCF with Smc3 at WT only, common and mutant only sites (Fig. 4C and Extended Data Fig. 4). In contrast to the data shown in the heatmaps (Fig. 4B), for this analysis we first removed any Smc3 sites that were not specific to WT binding at WT only, or mutant CTCF binding at mutant specific sites since those Smc3 sites are likely CTCF-independent (Extended Data Fig. 4). The data demonstrate that Smc3 overlaps with both WT and mutant CTCF at a significantly higher percentage at common sites (80–100%) compared to WT only and mutant only binding sites (below 55%), consistent with stronger and more accessible WT and mutant CTCF peaks at these locations (Fig. 4C). While we show that the percentage of common sites varies between mutants (Fig. 3 and Extended Data Fig. 3), the latter appear to retain their function to block cohesin at these accessible locations. Indeed, the percentage change between cohesin overlap for WT versus mutant CTCF varies little, however the number of cohesin-CTCF bound sites for each subset of WT only, common and mutant only bound sites can be considerably different as shown by the numbers of above each bar in Fig. 4C. This is also reflected by the numbers of SMC3-CTCF versus CTCF bound sites in Fig. 4B.

**Extended Data Fig. 5:**
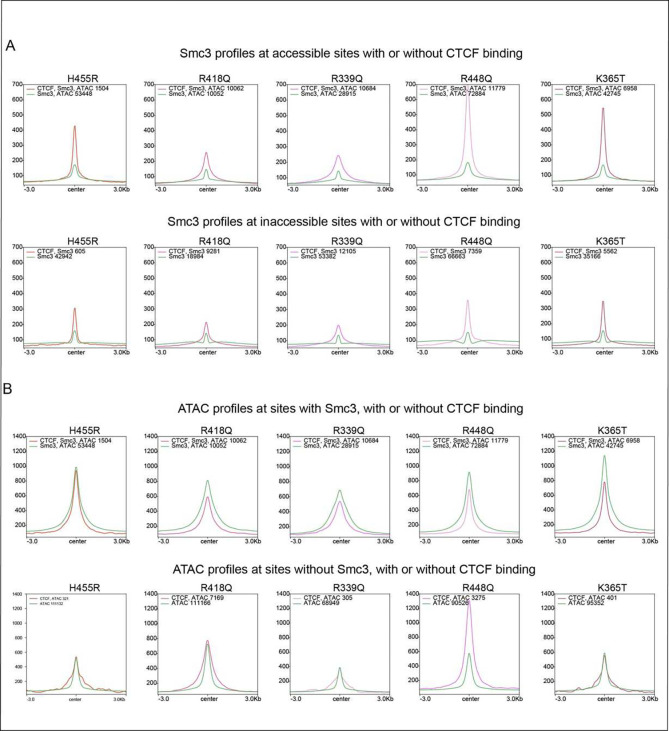
The relationship between CTCF binding, cohesin overlap and accessibility (**A)** Profiles of Smc3 binding in WT and mutant CTCF expressing cells is shown for accessible (top) and inaccessible (bottom) sites with (color-coded for each mutant) or without (green) CTCF binding. The remaining mutants are shown in Fig. 4D. (**B**) Profiles of ATAC-seq in WT and mutant CTCF expressing cells is shown for accessible Smc3 sites (top) and accessible sites without Smc3, with (color-coded for each mutant) or without (green) CTCF binding. The remaining mutants are shown in Fig. 4E. CTCF bound accessible sites have a narrower ATAC-seq peak compared to unbound sites at Smc3 sites. Each CTCF mutant has a differential ability to decrease the breadth of ATAC-seq peaks.

A comparison of Smc3 profiles at CTCF bound versus unbound sites demonstrates that other factors such as TFs which are responsible for accessibility, contribute very little to Smc3 signal compared to CTCF. Smc3 profiles at CTCF bound sites confirmed that CTCF is most functional at blocking cohesin at accessible sites and further demonstrate that each mutant CTCF performs this function with variable effectiveness, (Fig. 4D and Extended Data Fig. 5A). This trend is also observed at inaccessible Smc3 sites but with a lower overall Smc3 intensity in both WT and mutants, suggesting that CTCF bound at inaccessible sites is less likely to be associated with loop extrusion. These data suggest that for a given cell type, the subset of strong and accessible CTCF binding is predominantly involved in loop extrusion. However, mutant binding at *de novo* sites can be functional and block cohesin at low levels depending on the mutant and the level of accessibility at those locations (Fig. 4C, 3A and Extended Data Fig. 3).

### CTCF-dependent loop extrusion reduces ATAC-seq signal

To further investigate the link between accessibility and CTCF binding, we compared the ATAC-seq signal at CTCF bound versus unbound sites in the presence or absence of cohesin (Fig. 4E and Extended Data Fig. 5B). Surprisingly, while functional bound CTCF sites overlapping Smc3 showed strong ATAC-seq signals, accessibility was reduced when CTCF was bound compared to accessible Smc3 sites without CTCF. This was observed for both WT and mutant proteins. Each CTCF mutant exhibited a differential ability to decrease the breadth of ATAC-seq peaks mirroring its binding stability, suggesting that CTCF is driving the change in accessibility and that the relationship between CTCF and accessibility is bidirectional. A reduction in accessibility was only observed at CTCF bound sites overlapping Smc3. Sites lacking Smc3 are unable to form loops, lending support to the idea that loop formation decreases accessibility.

In sum, these results demonstrate the importance of identifying the molecular and epigenetic features that drive both CTCF site-specific and stable binding. Furthermore, they highlight the usefulness of the mutants in distinguishing cause from effect: without the mutants it would not be possible to conclude that binding of CTCF involved in loop extrusion drives changes in accessibility.

### Each CTCF mutation alters gene expression and TF binding in a unique manner.

**Fig. 5:**
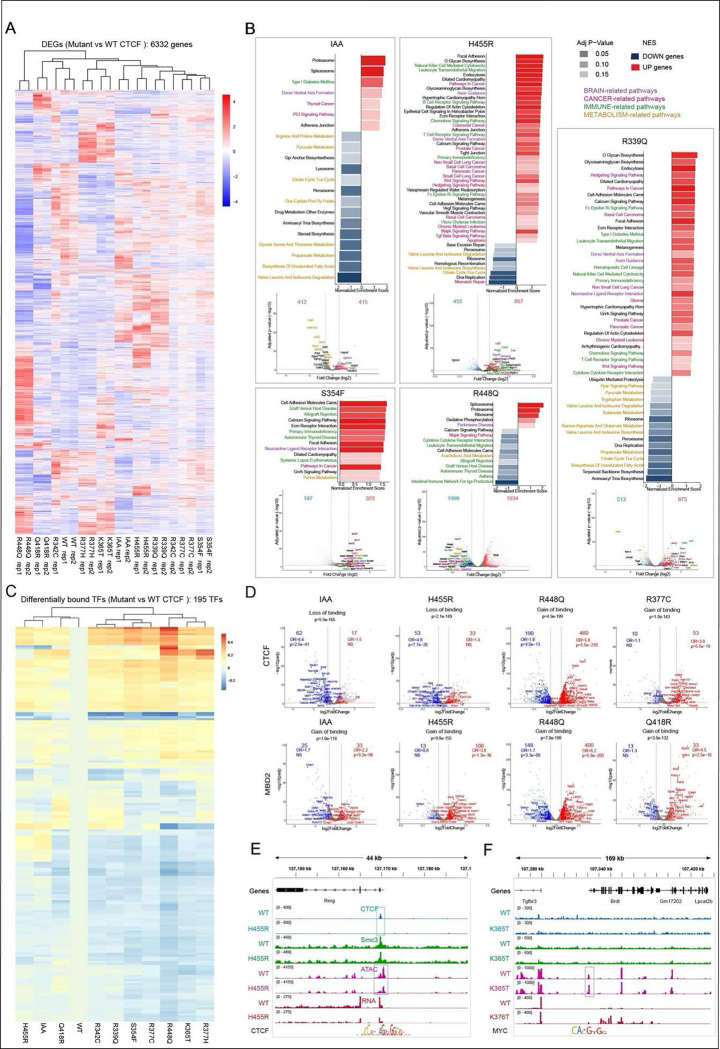
CTCF mutations alter gene expression and TF binding. (**A)** Heatmap showing unsupervised clustering of standardized and normalized expression levels of differentially expressed genes (DEGs) in cells that have endogenous CTCF degraded in the absence or presence of WT transgene or mutant CTCF transgenes. Genes are considered differentially expressed in comparison to cells expressing the WT CTCF transgene in the absence of endogenous CTCF. (**B**) Gene set enrichment analysis of DEGs. Examples shown are cells with endogenous CTCF degraded in the absence (IAA), and presence of the mutant CTCF transgenes, H455R, S354T, R448Q and R339Q. All other mutants are shown in Extended Data Fig. 6A. For each condition, bar graphs show significantly enriched KEGG pathways. The volcano plots below highlight the DEGs belonging to these enriched pathways with genes outside of these pathways shown in black. Brain related pathways are shown in purple, cancer in pink, immune in green and metabolism in mustard. (**C**) Heatmap showing differentially bound TFs (predicted from footprinting analysis of ATAC-seq data using the TOBIAS pipeline) in cells which have endogenous CTCF degraded in the absence (IAA) or presence of WT or mutant CTCF transgenes (ID condition). For this analysis replicates were merged and compared with cells expressing the WT CTCF transgene in the absence of endogenous CTCF. (**D**) Volcano plots show examples of two differentially bound TFS (CTCF, MYC) overlapping the promoters of DEGs in CTCF degraded (IAA) and CTCF mutant expressing cells (ID). The remaining mutant comparisons are shown in Extended Data Fig. 6B. The enrichment of the TF target genes among the up- and down-regulated genes are reported on top on the volcanos (Odds Ratios (ORs), and p-values). (**E,F**) Examples of altered CTCF binding at the promoter of differentially expressed *Rerg*, and predicted differentially bound MYC at the promoter of differentially expressed *Brdt*.

To determine how CTCF mutations impact gene expression we performed RNA-seq and did an unsupervised clustering analysis, comparing gene expression in cells in which endogenous CTCF was degraded in the absence (IAA) or presence of the WT or mutant CTCF transgenes (ID). Alterations in transcriptional output were determined by comparison with cells expressing WT transgenic CTCF (ID) (Fig. 5A). Overall, 6332 genes had altered gene expression across all mutants and including the IAA condition, a selection of which are labelled in the heatmap. As expected, H455R, which loses the most binding sites has the closest profile to the IAA condition. Also of note is the difference in gene expression changes between R377C and R377H, two distinct mutants of the same residue which give rise to different sets of differentially expressed genes (DEGs).

**Extended Data Fig. 6:**
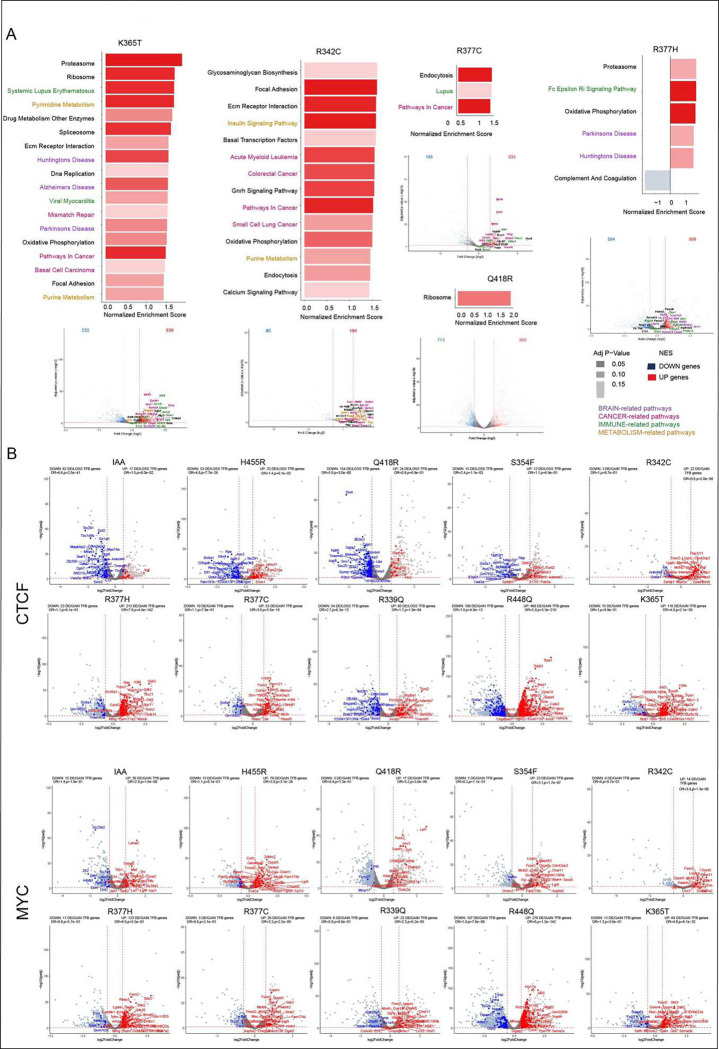
CTCF mutations alter gene expression and TF binding. (**A**) Gene set enrichment analysis of DEGs. All other mutants are shown in Fig. 5B. For each condition, bar graphs show significantly enriched KEGG pathways. The volcano plots below highlight the DEGs belonging to these enriched pathways with genes outside of these pathways shown in black. Brain related pathways are shown in purple, cancer in pink, immune in green and metabolism in mustard. (**B**) Volcano plots show examples of two differentially bound TFS (CTCF, MYC) overlapping the promoters of DEGs in CTCF mutant expressing cells (ID). The remaining mutant comparisons are shown in Fig. 5D. The enrichment of the TF target genes among the up- and down-regulated genes are reported on top on the volcanos (Odds Ratios (ORs), and p-values).

Gene set enrichment analysis using the KEGG database ^36^ showed a strong enrichment in functional pathways related to cancer, brain, immune and metabolic processes (color coded in Fig. 5B, with genes outside these pathways represented in black) among DEGs across mutants. This finding is compatible with the human diseases associated with CTCF mutations. The top differentially expressed genes of those pathways are shown in volcano plots below with the same color-code. Examples in Fig. 5B show changes in pathway and gene expression in the absence of CTCF (IAA condition) and in cells expressing the mutant CTCF ansgenes, H455R of ZF7, S354F of ZF4, R448Q of ZF7 and R339Q of ZF3 (ID condition). All other mutants are shown in Extended Data Fig. 6A.

The data in Figures 5A, B demonstrate that each mutant displays a distinct profile of gene expression changes linked to alterations in a unique set of functional pathways. Interestingly, cells in which endogenous CTCF is degraded (IAA condition) show fewer gene expression changes compared to mutant expressing cells, with the exception of the R377C mutation (Extended Data Fig. 6A). Some of the mutants have alterations in many pathways (R399Q of ZF3), while others highlight changes in only a few (R418Q of ZF6 and R377C of ZF4) as shown in Fig. 5B and Extended Data Fig. 6A, suggesting that for those mutants, the alteration in gene expression may not accumulate in specific pathways.

To investigate whether mutant specific transcriptional changes could be explained by altered TF binding, we performed a footprinting analysis of ATAC-seq data using the TOBIAS pipeline ^37^ in cells which have endogenous CTCF degraded in the absence (IAA) or presence of WT or mutant transgenes (ID). For this analysis replicates were merged and comparisons made with WT ID cells. Overall, 195 TFs had predicted differential binding across all mutants and including the IAA condition, a selection of which are labelled in the heatmap. As a proof of principle, we detected predicted differential binding for CTCF, which we know from our analyses has altered binding in the mutants, and consistent with the profiles in Figures 2, 3 and 4, we see the most depletion in the H455R mutant and the IAA condition and most enrichment in the R448Q mutant of ZF7. Additionally, the two R377 mutants give rise to distinct profiles of predicted differential CTCF binding. Numerous other predicted differentially bound TFs (n = 194) were identified as shown in Fig. 5C.

To analyze the impact of predicted differential binding of TFs on gene expression, we overlapped predicted differentially bound TFs with a region of 2kb around the promoters of DEGs in cells expressing different CTCF mutants. Although TF binding at gene promoters regulates gene expression, TFs can also bind distal and proximal enhancers to exert transcriptional control. However, since we cannot connect enhancers to their target genes with any certainty, these regulatory elements are excluded from our analysis and we focus only on DEGs with predicted differentially bound TFs at their promoters, where we observed a strong enrichment among the DEGs suggesting that part of the transcriptional changes associated with CTCF mutations could be explained by the disruption of specific TF pathways (**Supplementary Table 1**). Fig. 5D and Extended Data Fig. 6B highlight two predicted differentially bound TFs (CTCF and MBD2) at promoters of DEGs. These are shown in volcano plots for cells in which endogenous CTCF is degraded (IAA) and cells expressing different CTCF mutations (ID). Consistent with our finding that each CTCF mutation alters TF binding and gene expression in a unique manner, we show that for each CTCF mutant, predicted differentially bound TFs target the promoters of a distinct set of up (red) and down (blue) regulated genes. Upregulated genes could reflect enriched binding of a TF at a promoter if the factor acts as an activator, but we also cannot exclude the possibility that upregulation of expression could occur through depletion of a TF that acts as a silencer. Similarly, downregulated genes could reflect reduced or enriched binding of a TF at a promoter where a factor is respectively acting as an activator or repressor. In general, however, we find that predicted enrichment and depletion of binding of TFs is respectively associated overall with up and downregulation of gene expression (Fig. 5D and Extended Data Fig. 6B and **Supplementary Table 1**).

Examples of (predicted and validated) altered CTCF binding at the promoter of differentially expressed *Rerg*, and predicted differentially bound MYC at the promoter of differentially expressed *Brdt*, are shown in Fig. 5E, F. Loss of CTCF binding at the promoter of the *Rerg* gene leads to a decrease in its expression, while expression of *Brdt* increases in line with increased predicted binding of MYC at its promoter. The latter is an example of an indirect effect because there is no CTCF bound at this gene.

In sum, combined analysis of RNA-seq and ATAC-seq highlight the variable effects of each CTCF mutation, connecting gene expression changes with 195 predicted differentially bound TFs, to provide a deeper understanding of the factors that underlie the unique transcriptomic profile of each CTCF mutation.

### Chromatin interactivity and insulation are linked to mutant binding properties

**Fig. 6:**
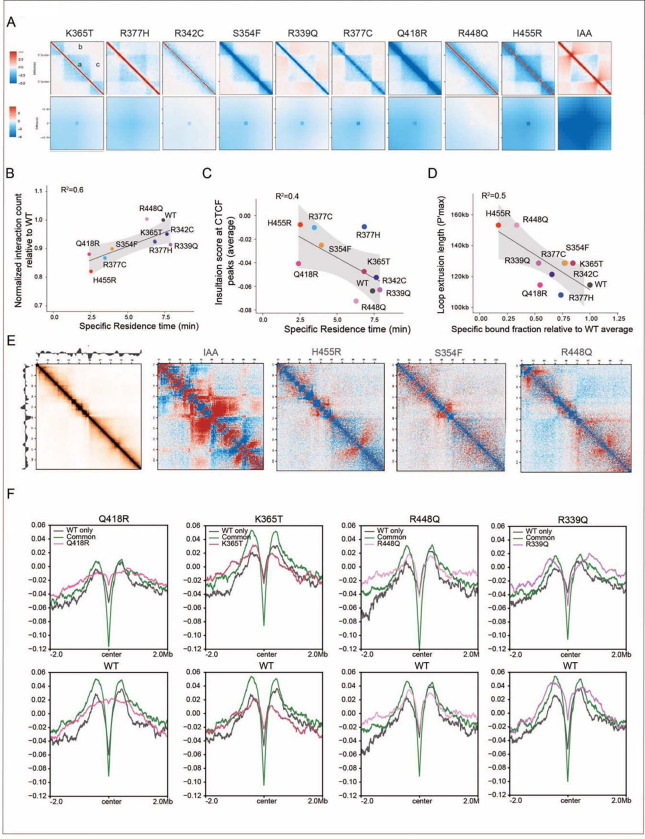
CTCF mutations alter chromatin interactivity. **(A)** Aggregated analyses. The top panels show the aggregated differential TAD analysis (ATA) for WT and each CTCF mutant. The middle square represents the aggregated TAD flanked by the upstream and downstream TAD**. (a)** Reflects the intra-TAD interaction, while **(b)** and **(c)** highlight the inter-TAD interactions**.** The lower panels show the aggregated differential peak analysis (APA). For the ATA, we performed a pairwise comparison using the union of the boundaries detected in WT and the corresponding mutant. For the APA, we performed a pairwise comparison using the union of loops found in WT and the corresponding mutant. The gain of interactions is color-coded in red and the loss of interactions in blue. These analyses were performed at 10 kb resolution. (**B**) Correlation between the normalized interaction count (at the aggregated loops as described in Fig. 6A) relative to WT estimated by Hi-C and the specific residence time detected by FRAP. These analyses were performed at 10 kb resolution. (**C**) Correlation between the insulation at score at CTCF peaks and the specific residence time detected by FRAP. (**D**) Correlation between the loop extrusion length estimated by the P’max values derived from Hi-C and the specific bound fraction detected by FRAP. These analyses were performed at 10 kb resolution. (**E**) Example of differential interactions between CTCF mutant and WT. The left matrix shows the interaction in WT within a 10 Mb region at 40 kb resolution. The graphs on the side of the matrix represent the insulation score. The differential matrices are shown for IAA, H455R, S354F and R448Q as examples. Gained interactions are color-coded in red and lost interactions in blue. (**F**) Profiles show the aggregated insulation score at WT only, common and mutant only binding sites for four mutants. The profiles for the remaining mutants are shown in Extended Data Fig. 7A.

To determine how CTCF mutants impact chromatin interactions we performed Hi-C. We found that each mutant has a distinct interaction profile and exhibits (i) variable loss of intra TAD interactions and boundary strength as well as (ii) aggregated loop strength. The IAA condition, in which CTCF is degraded shows a loss of intra TAD interactions and a gain of inter TAD interactions as well as the most reduction in aggregated loop strength (Fig. 6A), while mutants display their own unique effects on TAD interactions and loop strength.

Consistent with our finding that cohesin overlap is correlated with CTCF mutant residence time as determined by FRAP analysis (Fig. 4B), we found that residence time is also associated with chromatin interactivity and insulation score (Fig. 6B, C). Gassler et al., showed that the first derivative of the relative contact probability curve after log-log transformation (P’max) provides an estimate of the average loop size and cohesin density ^38^. Here we show that the loop extrusion length (P’max) for each mutant is associated with the fraction that is bound to chromatin (Fig. 6D), and the longest loops are associated with mutants that have the lowest bound fraction indicating a lower density of CTCF-cohesin anchors in these mutants (Fig. 4B). The variability in the interaction profile of each mutant can be clearly seen across a 10Mb region of chromosome (Fig. 6E).

To further examine the impact of each mutant on chromatin organization, we analyzed the aggregated insulation score at WT only, common and mutant only binding sites using a 2Mb window centered on CTCF peak summits (Fig. 6F). The insulation scores were generated from Hi-C data at a 10kb resolution. This analysis revealed that the strong, common accessible binding sites with the highest cohesin overlap (Fig. 4C), have the highest insulation (lowest insulation score) compared to WT only and mutant only sites. Whether insulation scores at WT only and mutant only sites are lower or higher than each other appears to depend on the residence time and cohesin overlap of each mutant, in line with what we observed in Fig. 4B, C and 6C. These studies further demonstrate that imaging and molecular analyses can be functionally integrated to provide new insight into mutant specific effects.

### Changes in gene expression are linked to changes in chromatin interactivity

**Fig. 7:**
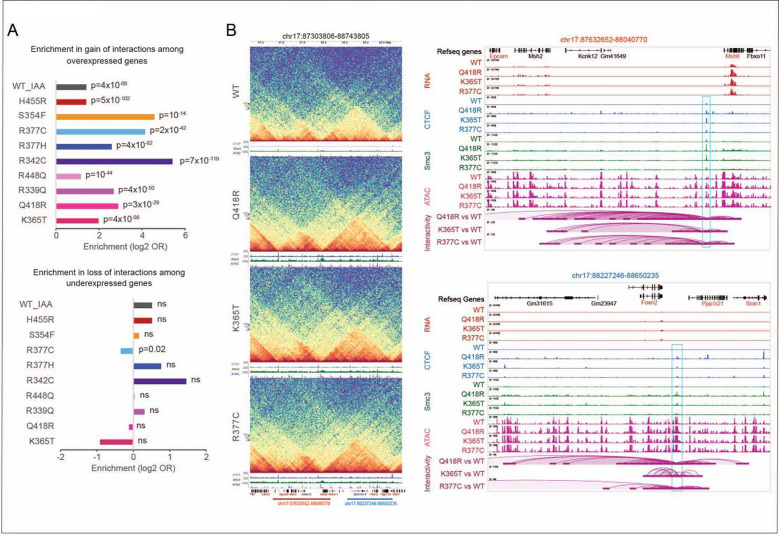
Changes in gene expression are linked to changes in chromatin interactivity: (**A**) Bar graphs showing the enrichment in over-expressed (top) or under-expressed (bottom) genes among gained (top) or lost (bottom) loops with one anchor overlapping the promoter of the DEGs in IAA and CTCF mutants. (**B**) Example of 2 loci (blue and red rectangles) with a direct effect of gain in CTCF binding and chromatin interactivity. The left panel shows the Hi-C interaction matrices in WT, Q418R, K365T and R377C with both gain of intra- and inter-TAD interactions in the mutant compared to WT. The left panels show the zoom-in tracks of CTCF (blue) Smc3 (green), ATAC (red) and significant differential chromatin loops (purple) with one anchor overlapping the differential CTCF binding sites at both loci. Overexpressed genes are highlighted in red. Of note, only gained interactions were detected at these loci. This analysis was performed at 10 kb resolution.

To determine if changes in gene expression are linked to altered chromatin folding, we analyzed interactions involving the promoters of genes that were differentially expressed. We found that the promoters of overexpressed genes were enriched in gain of chromatin loops (Fig. 7A). This enrichment was not observed among the under-expressed genes, suggesting that under-expression observed in mESC expressing mutant CTCF might be independent of CTCF’s function in chromatin organization, while overexpression might reflect a direct CTCF-mediated effect on loop extrusion as depicted in the examples showing the gain of interactions from CTCF binding sites toward the promoters of overexpressed genes, including *Msh6*, *Epcam*, *Foxn2* and *Cox7a2l* (Fig. 7B and **S7B**). Interestingly, all these genes are implicated in tumorigenesis. Indeed, MSH6 is a mismatch repair factor and its mutation is associated with Lynch syndrome and cancer susceptibility ^39^. Overexpression of MSH6 might also exert an oncogenic function in glioblastoma by promoting cell proliferation ^40^. *Epcam* encodes for a membrane glycoprotein involved in epithelial cell adhesion and has been described as both an oncogene and tumor suppressor depending on the microenvironment ^41^. FOXN2 acts as a tumor suppressor in multiple cancers, including breast, lung and liver ^42–44^. Overexpression of COX7A2L might promote hypoxia tolerance in breast and endometrial cancer ^45^.

## DISCUSSION

Using a combination of imaging, structural and molecular approaches we have examined the impact of nine, high frequency cancer associated CTCF mutations in ZFs that make contact with the core consensus binding motif. Collectively the graded mutant perturbations offer a platform for investigating the molecular and epigenetic features that govern CTCF’s choice of binding sites, and the degree of stability with which CTCF binds at individual locations and is able to function as a chromatin organizer. Since the mutants allow us to distinguish direct from indirect CTCF-mediated effects and cause from consequence, they provide a deeper understanding of CTCF’s downstream impact on chromatin and gene expression.

Our studies reveal that the specificity of interaction between the ZFs in CTCFs binding domain and its target DNA sequence is a key determinant of attachment. However, if the same sequence is found in both accessible and inaccessible chromatin, CTCF will preferentially bind the accessible sites as highlighted by the fact that mutants bind common accessible sites in preference to less accessible WT only sites. Thus, sites in open chromatin dominate the search space. This is underscored by the strong correlation between the fraction of chromatin bound mutants detected by FRAP, and mutant binding at common, accessible CTCF sites. We also observed a good correlation between each mutant’s residence time and CTCF-cohesin overlap, suggesting a model in which binding stability is important for blocking cohesin movement on chromatin. A similar correlation was found between residence time and chromatin interactivity as well as insulation score, linking binding stability with cohesin overlap as well as loop and boundary formation.

In principle, these features could have been uncovered by analyzing WT CTCF in the context of cohesin overlap and accessibility. However, collectively the graded effects of the mutants act as perturbations that lend support to our models, underscoring how different aspects of CTCFs binding properties contribute. Moreover, they reveal that each mutant’s search for binding sites can be decoupled from its chromatin bound residence time, which is a reflection of its binding stability. For example, the Q418R mutant binds an intermediate number of sites but at these sites it is has a very low residence time and is less competent at blocking cohesin. In contrast, R448Q binds fewer sites with a similar residence time compared to WT CTCF, and it is slightly better at blocking cohesin than WT protein. Furthermore, H455R and R448Q, which have very different chromatin residence times, have similarly low chromatin bound fractions, correlated to their P’-max. The increased loop length of both mutants can be explained by the unobstructed passage of cohesin past what would normally be a bound CTCF site.

The crosstalk between accessibility and CTCF binding goes in both directions, such that accessibility affects binding and binding affects accessibility. Although binding at inaccessible sites has little impact on ATAC-seq signal, binding at accessible sites, narrows and reduces the ATAC-seq peak. Evidence for this being a CTCF driven effect comes from analysis of the CTCF mutants. Those with low binding stability (H455R and Q418R) are unable to function in this capacity, while the R342C mutant has a stronger effect compared to WT CTCF. We speculate that these differences reflect decreased accessibility at the bases of CTCF-Smc3-mediated loops, because in the absence of Smc3, when loops cannot form there is no reduction in accessibility.

While loss or gain of CTCF binding at promoters can account for direct mutant specific effects on accessibility and gene expression, we could only demonstrate a direct effect of gained CTCF binding associated with gained loops involving the promoters of over-expressed genes. Under-expression could, therefore, more often reflect indirect effects of CTCF binding alterations. Indeed, all mutants have their own unique indirect impact in globally changing accessibility at sites where CTCF is not bound. This effect, links changes in TF footprinting with altered transcriptional output, explaining some of the mutant specific effects that we observed.

Aside from the R377C mutant, we observed fewer changes in gene expression in IAA treated cells compared to cells expressing CTCF mutants. Although the mutants all uniquely affect gene expression pathways, we detected enrichment in pathways affecting the brain, immune system, cancer and metabolism, which is consistent with the clinical setting in which the mutations are found, namely cancer and brain disorders. The added effect on immune and metabolic pathways provides insight into other changes that could occur in CTCF mutant-mediated diseases.

Taken together the binding domain mutants we have analyzed here, provide a new appreciation of CTCF’s bidirectional relationship with chromatin, its ability to bind and function in different contexts as well as the potential impact of each mutation in clinical settings. Furthermore, our analyses provide a better understanding of how any genetic or epigenetic disorder that alters the landscape of chromatin can in turn impact CTCF binding and function.

## Methods

### Cell lines

Mouse embryonic stem cells E14Tg2a (karyotype 19, XY; 129/Ola isogenic background) and all clones derived from these were cultured under feeder-free conditions in 0.1% gelatin (Sigma ES-006-B) coated dishes (Falcon, 353003) at 37°C and 5% CO_2_ in a humidified incubator. The cells were grown in DMEM (Thermo Fisher, 11965–118) supplemented with 15% fetal bovine serum (Thermo Fisher, SH30071.03), 100 U/ml penicillin - 100 μg/ml streptomycin (Sigma, P4458), 1 X GlutaMax supplement (Thermo Fisher, 35050–061), 1 mM sodium pyruvate (Thermo Fisher, 11360–070), 1 X MEM non-essential amino-acids (Thermo Fisher, 11140–50), 50 μM b-mercaptoethanol (Sigma, 38171), 10^4^ U/ml leukemia inhibitory factor (Millipore, ESG1107), 3 μM CHIR99021 (Sigma, SML1046) and 1 μM MEK inhibitor PD0325901 (Sigma, PZ0162). The cells were passaged every alternate day by dissociation with TrypLE (Thermo Fisher, 12563011).

### DNA constructs

Construction of vector for cloning transgenic, doxycycline-inducible expression of WT and mutant mouse *Ctcf* cDNA were obtained from GenScript in pUC19 vectors. The cDNA was amplified such that it harbors an AflII sequence at the 3’ end of the gene and fused to a FLAG tag (that harbors NotI sequence) at the 5’ end with the help of a fusion PCR. The resultant fragment was digested with NotI and AflII. The *Ctcf* gene was removed from pEN366 ^4^ by digesting with the same enzymes. This backbone was used for insertion of each *Ctcf* mutant.

In brief, the cDNA region corresponding to each of the C and N terminals and zinc fingers were PCR amplified in such a way that it included a short stretch of the 5′ and/or 3′ region of the neighboring fragment to be connected. The desired PCR products were then annealed, amplified by PCR and cloned into the NotI and AflII sites of the pEN366 backbone (Addgene #156432). All of the constructs were verified by DNA sequence analysis. For all transgenes, the final vector harbors an N terminal 3 X FLAG tag and a C terminal *mRuby* as in-frame fusion to WT and mutant *Ctcf*. The vector also harbors a *TetO-3G* element and *rtTA3G* for doxycycline induced expression of the transgene, and homology arms surrounding the sgRNA target site of the *Tigre* locus for locus-specific insertion. The selection of stable integrants was achieved by virtue of *FRT-PGK-puro-FRT* cassette. Further details of the vector are described elsewhere ^4^. The vector pX330-EN1201 ^4^ harboring spCas9 nuclease and *Tigre*-targeting sgRNA was used for targeting of *Tigre* locus (Addgene #92144).

### Gene targeting

Mouse embryonic stem cell E14Tg2a harboring *Ctcf-AID-eGFP* on both alleles and a knock-in of pEN114 - *pCAGGS-Tir1-V5-BpA-Frt-PGK-EM7-PuroR-bpA-Frt-Rosa26* at *Rosa26* locus was used as the parental cell line for making all the transgenes ^4^. pEN366 derived vectors harboring the rescue transgenes (WT and mutant *Ctcf*) were used for targeting transgenes to the *Tigre* locus (clone ID# EN156.3.5) ^8^. For nucleofections, 15 μg each of plasmids harboring the transgenes and 2.5 μg of those with sgRNA targeting the *Tigre* locus was used. Nucleofection were performed using Amaxa P3 Primary Cell kit (Lonza, V4XP-3024) and 4D- transfector. 2 million cells were transfected with program CG-104 in each case. The cells were recovered for 48 h with no antibiotic followed by selection in puromycin (1 μg/mL) (Thermo Fisher, A1113803). Single colonies were manually picked and expanded in 96 well plates. Clones were genotyped by PCR and FACS was performed to confirm that the level of expression of transgenes were comparable. All the clones that were used for the analyses were homozygous for the integration of the transgenes and their levels of expression were comparable.

### Induction of auxin inducible degradation of CTCF and doxycycline induced expression

For degradation of endogenous CTCF, the auxin-inducible degron was induced by adding 500 μM indole-3-acetic acid (IAA, chemical analog of auxin) (Sigma, I5148) to the media. Expression of transgenes was achieved by the addition of doxycycline (Dox, 1 μg/ml) (Sigma, D9891) to the media. The cells were treated with IAA and/or Dox for 2 days.

### Western Blotting

mESCs were dissociated using TrypLE, washed in PBS, pelleted and used for western blotting. Approximately 2 million cells were used to prepare cell extract. Cell pellets were resuspended in RIPA lysis buffer (Thermo Fisher, 89900) with 1X HALT protease inhibitors (Thermo Fisher, 78430), incubated on ice for 30 min, spun at 4°C at 13,000 rpm for 10 min and supernatant was collected. For the western blot of CTCF, low salt lysis buffer (0.1 M NaCl, 25 mM HEPES, 1 mM MgCl_2_, 0.2 mM EDTA and 0.5% NP40) was used supplemented with 125 U/ml of benzonase (Sigma E1014). Protein concentration was measured using the Pierce BCA assay kit (Thermo Fisher, 23225). 20 μg of protein were mixed with Laemmli buffer (Biorad, 1610737) and β-mercaptoethanol, heated at 95°C for 10 min and run on a Mini-protean TGX 4%−20% polyacrylamide gel (Biorad, 456–1095). The proteins were transferred onto PVDF membranes using the Mini Trans-Blot Electrophoretic Transfer Cell (Bio-Rad, 170–3930) at 80 V, 120 mA for 90 min. PVDF membranes were blocked with 5% BSA in 1 X TBST prior to the addition of antibody. The membranes were probed with appropriate antibodies overnight at 4°C (anti-rabbit histone H3 (abcam, ab1791; 1: 2,500 dilution), anti-mouse FLAG antibody (Sigma, F1804; 1: 1,000 dilution), anti CTCF (active motif, 61311), anti Rad21 (ab992). Membranes were washed five times in PBST (1 × PBS and 0.1% Tween 20) for 5 min each and incubated with respective secondary antibodies in 5% BSA at room temperature for 1 h. The blots were rinsed in PBST and developed using enhanced chemiluminescence (ECL) and imaged by Odyssey LiCor Imager (Kindle Bioscien ces).

### Flow cytometric analysis

Cells were dissociated with TrypLE, washed and resuspended in MACS buffer for flow cytometric analysis on LSRII UV (BD Biosciences). Analysis was performed using the FlowJo software.

### Cell culture – FRAP

Control cells expressing H2B-mRuby were engineered using the same background as the CTCF transgene expressing cells, E14Tg2a mESCs. Briefly, pEN114 - *pCAGGS-Tir1-V5-BpA-Frt-PGK-EM7-PuroR-bpA-Frt-Rosa26* was used as the parental cell line to be nucleofected by the pASH40-mRuby-neo with the PiggyBac transposase vector. For the FRAP experiment, CTCF and H2B expressing cells were cultured for two days on 35 mm no 1.5H glass-bottom imaging dishes (MatTek, Ashland, MA, P35G-1.5–14C) coated with Geltrex (Gibco, A1413201) according to manufacturer’s instructions. 48 hours prior to the experiment, the culture medium was changed to medium supplemented with 500 μM 3-Indoleacetic acid (IAA; Sigma-Aldrich, I2886–5G) and 1 μg/mL doxycycline (Sigma-Aldrich, D3072–1ML). Just prior to imaging, the medium was changed to imaging medium: phenol red free DMEM with all other aspects of the medium the same.

### Fluorescence recovery after photobleaching (FRAP)

FRAP experiments were performed on an LSM900 confocal microscope (Zeiss, Germany) equipped with a full, humidified incubation chamber that was maintained at 37°C with 5.5% CO_2_. The time series were acquired in confocal mode using two-stage acquisition with the following excitation parameters: 561nm excitation laser at 0.8% power, 53μm pinhole size corresponding to 1AU, scan speed 7, and 3x crop factor corresponding to a pixel dwell time of 3.06μs. During the first phase, we imaged 25 frames with 0.5 seconds between frames, and during the second phase we imaged 160 frames with 4 seconds between frames, resulting in a total movie length of 657 seconds. For bleaching, a 1μm diameter ROI was selected. Bleaching was performed after frame 8 using both the 488nm and 561nm lasers at 100% power, 53μm pinhole size, and scan speed 7 corresponding to a pixel dwell time of 3.06μs. For each condition, 30 movies were acquired and between 3 and 11 were excluded due to drift.

### Fluorescence recovery after photobleaching (FRAP) analysis

FRAP analysis was performed using custom Python scripts. Briefly, movies were loaded into a custom Python GUI (https://github.com/ahansenlab/FRAP-drift-correction-GUI). The bleached nucleus was segmented using manually selected segmentation parameters, and was used to estimate $I_{nonbleach}(t)$ – the average nuclear intensity – and $I_{background}(t)$ – the average background intensity. To correct for cell drift, the ROI positions were manually updated at a number of frames and the ROI position was linearly interpolated between manual updates. Cells with excessive drift were manually excluded and the mean fluorescence intensity of the bleach – $I_{bleach}(t)$ – was taken as the average intensity within the ROI.

To correct for photobleaching, $I_{nonbleach}(t)$ was smoothed using an averaging filter, and a series of correction factors (C(t)) were computed as the ratio of these values to their average prebleach value as follows:

$$C(t)=\frac{I_{nonbleach,smooth\;}(t)\;-\;I_{background\;}(t)}{{\left\langle I_{nonbleach\;}(t)\;-\;I_{background\;}(t)\right\rangle }_{prebleach\;}}$$

Subsequently, the FRAP curves were normalized according to the following equation:

$$\underline{I_{bleach}(t)}=\frac{C(t)\left(I_{bleach}(t)\;-\;I_{background\;}(t)\right)}{{\left\langle C(t)\left(I_{bleach\;}(t)\;-\;I_{background\;}(t)\right)\right\rangle }_{prebleach\;}}$$

where $\underline{I_{bleach}(t)}$ is the normalized, background-subtracted, and photobleaching corrected FRAP signal.

Finally, since previous measurements of CTCF’s free diffusion coefficient have indicated a reaction-dominant model is most appropriate, we fit the following, previously demonstrated model(Sprague, Pego et al. 2004, Hansen, Pustova et al. 2017).

$$FRAP(t)=1-C_{eq,fast}e^{-k_{off,fast}t}-C_{eq,slow}e^{-k_{off,slow}t}$$

where $k_{off,slow}<k_{off,fast}.\;C_{eq,slow}$ was taken as the specific bound fraction and the specific residence time was taken as $\frac{1}{k_{off,stow}}$. To estimate parameter distributions, we implemented a bootstrapping routine in which a set of 16 movies were randomly resampled from the full dataset 2500 times with replacement. For each sample, the above equation was fit and the parameter values were taken as one data point. The 2500 resampled points were then used as distributions for plotting the specific bound fraction and residence time. For ease of comparison, the specific bound fractions were normalized to the average WT value when plotting.

### ChIPmentation

mESCs were dissociated using TrypLE (Thermofisher #12605010) and washed once in 1X PBS. After counting, cells were divided in 10 million aliquots and resuspended in fresh 1X PBS (1million cells/1ml). For double cross linking 25mM EGS (ethylene glycol bis(succinimidyl succinate); Thermofisher #21565) were added and cells were put in rotation for 30 min at room temperature, followed by addition of 1% formaldehyde (Tousimis #1008A) for 10 min also in rotation at room temperature. Quenching was performed by adding glycine to a final concentration of 0.125M followed by incubations of 5 min at room temperature in rotation. Fixed cells were washed twice with 5ml of 1X PBS containing 0.5% BSA and centrifuged at 3000rpm for 5 min at 4°C. Pellets were finally resuspended in 500ul 1X PBS containing 0.5% BSA, transferred to 1.5ml Eppendorf and centrifuged at 3000rpm for 3 min at 4°C. Supernatant was completely removed, pellets were snap-frozen in liquid nitrogen and stored at −80°C. Fixed cells (10 million) were thawed on ice, resuspended in 350 μl ice cold lysis buffer (10 mM Tris-HCl (pH 8.0), 100 mM NaCl, 1 mM EDTA (pH 8.0), 0.5 mM EGTA (pH 8.0), 0.1% sodium deoxycholate, 0.5% N-lauroysarcosine and 1X protease inhibitors) and lysed for 10 min by rotating at 4° C. Chromatin was sheared using a bioruptor (Diagenode) for 15 minutes ( 30 sec on, 30 sec off, high output level). 100ul of cold lysis buffer and 50ul of 10% Triton X-100 (f inal concentration of 1) were then added and the samples were centrifuged for 5 min at full speed at 4°C. Supernatant was collected, transferred to a new tube (Protein Low Binding tube) and shearing was continued for another 10 min, then the chromatin was quantified. FLAG M2 Magnetic Beads (Sigma, M8823) were used for FLAG immunoprecipitation. In other cases (CTCF, Cohesin, IgG) antibodies were bound to protein A magnetic beads by incubation on a rotator for one hour at room temperature. 10 μl each of antibody was bound to 50 μl of protein-A magnetic beads (Dynabeads) and added to the sonicated chromatin for immunoprecipitation at 4°C overnight. Next day, samples were washed and tagmentation were performed as per the original ChIPmentation protocol (Schmidl et al., 2015). In short, the beads were washed successively twice in 500 μl cold low-salt wash buffer (20 mM Tris-HCl (pH 7.5), 150 mM NaCl, 2 mM EDTA (pH 8.0), 0.1% SDS, 1% tritonX-100), twice in 500 μl cold LiCl-containing wash buffer (10 mM Tris-HCl (pH 8.0), 250 mM LiCl, 1 mM EDTA (pH 8.0), 1% triton X-100, 0.7% sodium deoxycholate) and twice in 500 μl cold 10 mM cold Tris-Cl (pH 8.0) to remove detergent, salts and EDTA. Subsequently, the beads were resuspended in 25 μl of the freshly prepared tagmentation reaction buffer (10 mM Tris-HCl (pH 8.0), 5 mM MgCl2, 10% dimethylformamide) and 1 μl Tagment DNA Enzyme from the Nextera DNA Sample Prep Kit (Illumina #20034198 ) and incubated at 37°C for 10 min in a thermomixer. Following tagmentation, the beads were washed successively twice in 500 μl cold low-salt wash buffer (20 mM Tris-HCl (pH 7.5), 150 mM NaCl, 2 mM EDTA (pH8.0), 0.1% SDS, 1% triton X-100) and twice in 500 μl cold Tris-EDTA-Tween buffer (0.2% tween, 10 mM Tris-HCl (pH 8.0), 1 mM EDTA (pH 8.0)). Chromatin was eluted and de-crosslinked by adding 70 μl of freshly prepared elution buffer (0.5% SDS, 300 mM NaCl, 5 mM EDTA (pH 8.0), 10 mM Tris-HCl (pH 8.0) and 10 ug/ml proteinase K for 1 hour at 55°C 850rpm and overnight at 65°C 850rpm. Next day, the supernatant was collected, transferred to new DNA Low Binding tubes and supplemented with an additional 30 μl of elution buffer. DNA was purified using MinElute Reaction Cleanup Kit (Qiagen #28204) and eluted in 20 ul. Purified DNA (20 μl) was amplified as per the ChIPmentation protocol (Schmidl, Rendeiro et al. 2015) using indexed and non-indexed primers and NEBNext High-Fidelity 2X PCR Master Mix (NEB M0541) in a thermomixer with the following program: 72°C for 5 m; 98°C for 30 s; 14 cycles of 98°C for 10 s, 63°C for 30 s, 72°C for 30 s and a final elongation at 72°C for 1 m. DNA was purified using Agencourt AMPure XP beads (Beckman, A63881) to remove fragments larger than 700 bp as well as the primer dimers. Library quality and quantity were estimatred using Tapestation (Agilent High Sensitivity D1000 ScreenTape #5067–5584 and High Sensitivity D1000 reagents #5067–5585) and quantified by Qubit (Life Technologies Qubit^™^ 1X dsDNA High Sensitivity (HS) #Q33230). Libraries were then sequenced with the Novaseq6000 Illumina technology according to the standard protocols and with around 200 million 150bp paired-end total per sample.

### Structural analyses of CTCF mutants

To complement the molecular analysis of CTCF mutants, the computational protein structure modeling of the CTCF protein was generated for the 8 mutated residues in ZF3-ZF7 under investigation. The potential effects of the 8 missense mutants were examined in the context of available CTCF protein structures, including the core DNA binding domain containing fragments of ZF1-ZF7 (PDB 8SSS), K365T (PDB 8SST), ZF2-ZF7 (PDB 5TOU), ZF3-ZF7 (PDB 5KKQ and 5T00), ZF4-ZF7 (PDB 5K5H), ZF5-ZF8 (PDB 5K5I and 5K5J), ZF6–8 (PDB 5K5L) and ZF4-ZF9 (PDB 5UND) in complex with cognate DNA (Hashimoto, Wang et al. 2017). Substitutions and side chain adjustments were made in PyMOL version 2.5.2 (Schrödinger, LLC), which was further used for the production of molecular graphics (Moore, Rabaia et al. 2012, Rosignoli and Paiardini 2022).

### Total RNA-seq

mESCs were dissociated using TrypLE, washed in 1X PBS, pelletted and 2.5 million cells were used for extracting RNA with RNeasy plus kit (Qiagen #74134) in each case. RNA quality was checked in Tapestation (Agilent High Sensitivity RNA ScreenTape #5067–5579, High Sensitivity RNA Sample Buffer #5067–5580 and High Sensitivity RNA Ladder #5067–5581) and quantified by Nanodrop. 500ng were used for Total RNA libraries preparation using Illumina kit (Illumina Stranded Total RNA Prep, Ligation with Ribo-Zero Plus #20040529). Library concentrations were estimated using Tapestation (Agilent High Sensitivity D1000 ScreenTape #5067–5584 and High Sensitivity D1000 reagents #5067–5585) and quantified by Qubit (Life Technologies Qubit^™^ 1X dsDNA High Sensitivity (HS) #Q33230). Libraries were then sequenced with the Novaseq6000 Illumina technology according to the standard protocols and with around 100 million 150bp paired-end total per sample.

### Hi-C

Hi-C was performed in duplicates using around 1 million cells each. mESCs were dissociated using TrypLE (Thermofishe #12605010), washed once in 1X PBS and resuspended in fresh 1X PBS (1million cells/1ml). For double cross linking 25mM EGS (ethylene glycol bis(succinimidyl succinate); Thermofisher #21565) were added and cells were put in rotation for 30 min at room temperature, followed by addition of 1% formaldehyde (Tousimis #1008A) for 10 min also in rotation at room temperature. Quenching was performed by adding glycine to a final concentration of 0.125M followed by incubations of 5 min at room temperature in rotation. Fixed cells were washed twice with 5ml of 1X PBS containing 0.5% BSA and centrifuged at 3000rpm for 5 min at 4°C. Pellets were finally resuspended in 500ul 1X PBS containing 0.5% BSA, transferred to 1.5ml Eppendorf and centrifuged at 3000rpm for 3 min at 4°C. Supernatant was completely removed, pellets were snap-frozen in liquid nitrogen and stored at −80°C. Samples were subsequently processed using the Arima Hi-C kit as per the manufacturer’s protocol and sequenced with the Novaseq6000 Illumina technology according to the standard protocols and with around 600 million 150bp paired-end reads per sample.

### ATAC-seq

We used an improved ATAC-seq protocol (Omni-ATAC) adopted from Corces et al., ^35^ with small adaptations. Briefly, cells in culture were treated with 200 U/ml DNase (Worthington # LS002007) for 30 min at 37°C to remove free-floating DNA and any DNA from dead cells. Cells were then harvested via trypsinization and resuspended in regular medium. After counting, 500,000 cells were collected, resuspended in 1X cold PBS and spin down at 500g for 5 min at 4°C in a fixed-angle centrifuge. After centrifugation, the supernatant was removed and the pellet resuspended in 500μl of cold ATAC-seq resuspension buffer 1 (10 mM Tris-HCl pH 7.4, 10 mM NaCl, 3 mM MgCl_2_, 0.1% NP-40 and 0.1% Tween-20 in water). 50ul (50.000 cells) were transferred to a new 1.5ml Eppendorf tube and 0.5ul 1% Digitonin (Promega #G9441) was added by pipetting well few times. The cell lysis reaction was incubated on ice for 3 min. After lysis, 1 ml of cold ATAC-seq resuspension buffer 2 (10 mM Tris-HCl pH 7.4, 10 mM NaCl, 3 mM MgCl_2_ and 0.1% Tween-20 in water) was added. Tube was inverted three times to mix and nuclei were pelleted by centrifugation for 10 min at 500g at 4°C. Supernatant was very carefully removed (pellet could be almost invisible at this step) and nuclei resuspended in 45μl of transposition mix (25μl 2X TD buffer (Illumina #20034198), 16.5μl 1X PBS, 0.5μl 1% digitonin, 0.5μl 10% Tween-20 and 2.5μl water) by pipetting up and down few times. 5ul of transposase enzyme (Illumina #20034198) was then added and the transposition reaction was incubated at 37°C for 30 min in a thermomixer with shaking at 1000 rpm. Reaction was cleaned up with Zymo DNA Clean and Concentrator-5 kit (Zymo#11–302C). Transposed DNA fragments were then amplified as described previously in Buenrostro et al., ^46^. Final libraries were purified with with Zymo DNA Clean and Concentrator-5 kit (Zymo#11–302C; Note: better to use two separate kits for pre- and post-amplification clean up), checked by Tapestation (Agilent High Sensitivity D1000 ScreenTape #5067–5584 and High Sensitivity D1000 reagents #5067–5585) and quantified by Qubit (Life Technologies Qubit^™^ 1X dsDNA High Sensitivity (HS) #Q33230). Libraries were then sequenced with the Novaseq6000 Illumina technology according to the standard protocols and with around 50 million 150bp paired-end reads per sample.

## QUANTIFICATION AND STATISTICAL ANALYSIS

### ChIP-seq data processing and quality control

Data were processed using Seq-N-Slide pipeline ^47^. Briefly, after trimming for NEXTERA adaptor sequence using trimGalore ^48^. The reads were aligned to mm10 genome with Bowtie2 ^49^. Ambiguous reads were filtered to use uniquely mapped reads in the downstream analysis. PCR duplicates were removed using Sambamba ^50^). Narrow peaks were called using MACS2 ^51^ in pair-end mode and with IgG as control for Smc3 ChIP-seq or WT untreated condition (in which FLAG CTCF is not expressed) for CTCF FLAG to control for unspecific binding, Peaks overlapping ENCODE blacklisted regions were filtered out ^52^. These procedures allowed us to minimize the false positive calls, as reflected by the high percentage of called peaks containing CTCF consensus motifs as detected by MEME ^34^ with 63% for WT. To ensure comparability between conditions, FLAG and SMC3 chip-seq samples were down-sampled to the same number of aligned (100 millions reads per replicates), except for 2 samples R377H-rep1 and R339Q-rep which were under-sequenced (with 68 and 90 millions reads, respectively) and good quality reads before peak calling using samtools ^53^. Bigwigs were obtained for visualization on individual as well as merged bam files using Deeptools ^54^. For CTCF FLAG visualization, the differential signals were generated after subtracting non-specific signal present in the WT untreated condition. Heatmaps and average profiles were performed on merged bigwig files using Deeptools.

### RNA-seq data processing and quality control

Data were processed using Seq-N-Slide pipeline ^47^. Briefly, after trimming for the Illumina adaptor and the T overhang as per Illumina recommendation using TrimGalore ^48^, reads were aligned against the mouse reference genome (mm10) using the STAR ^55^ aligner and differentially expressed genes were called using DESeq2 ^56^ with an adjusted p-value of 0.05 and a fold change cutoff of 1.5. Heatmap was performed using pheatmap R package ^57^and GSEA using fgsea R package ^58^and KEGG genesets ^36^.

### ATAC-seq data processing and quality control

Data were processed using Seq-N-Slide pipeline ^47^. Briefly, Reads were aligned to mm10 genome with Bowtie2 ^49^. Ambiguous reads were filtered to use uniquely mapped reads in the downstream analysis. PCR duplicates were removed using Sambamba ^50^. Mitochondrial contamination was assessed using samtools ^53^. All the samples showed less than 0.6% of mitochondrial DNA, Peaks were called using MACS2 ^51^and peaks overlapping ENCODE blacklisted regions were filtered out ^52^. Bigwigs were obtained for visualization on individual as well as merged bam files using Deeptools ^54^. Heatmaps and average profiles were performed on merged bigwig files using Deeptools.

### Hi-C Processing and Quality Control.

HiC-Pro ^59^ was used to align and filter the Hi-C data. To generate Hi-C filtered contact matrices, the Hi-C reads were aligned against the mouse reference genome (mm10) by bowtie2 (version 2.3.1) using local mode for the second step of HiC-Pro alignment (--very-sensitive -L 20 --score-min G,20,8 --local) to ignore the molecular barcode UMI sequences and dark bases introduced by Arima library prep kit at the 5’ end of the read. Singleton, ambiguous and duplicated reads were filtered out. Ligation sites for Arima HiC kit were set up using GATCGATC,GANTGATC,GANTANTC,GATCANTC. Low mapping quality (MAPQ<30), self circle, dangling ends and re-ligation reads were filtered out through the HiC-pro pipeline. Valid pairs represented more than 80% for all the samples. Samples were downscaled to the same number of good quality uniquely aligned reads using HiCExplorer ^60^. Of note, we did not use the number of valid pairs to downscale the samples to not overcorrect some conditions such as IAA and mutant H455R which were expected to show a dramatic biological decrease in valid interactions.

Downscaled interaction matrices were generated separately for each replicate and merged for visualization and downprocessing as mcool files using cooler ^61^. Samples were balanced using the ICE correction method ^62^ with HiCExplorer ^60^. HiC maps were generated from the merged downscaled and corrected matrices using GENOVA and HiCExplorer ^41,60^. RCP (relative contact probabilities) and insulation score were generated using GENOVA ^41^. After log-log transformation and Loess smoothing using R, the first derivative (P’max) was estimated as described in Gassler et al., ^38^.

## DOWNSTREAM ANALYSIS:

### CTCF peak motif analysis

Motifs were called using MEME ^34^ with a 1^st^ order background model on CTCF peaks called using MACS2 after separating the peaks into 3 groups for each pairwise comparison between WT and mutant CTCF: WT only, common and mutant only peaks. Peak intersection sets were generated using the intervene package ^63^and motifs were searched within 100 bp of each peak summit.

### ATAC-seq footprinting analysis

Footprinting analysis was performed on ATAC-seq signal using TOBIAS ^37^. Briefly, ATAC-seq signals were first corrected for Tn5 cutting sequence bias using the ATACorrect function and footprinting score calculated using FootprintScores at peaks detected by MACS. Finally differential footprinting estimated pariwise between WT and mutant CTCF using BINDetect and HOCOMOCO v11 core motif probability weight matrices ^64^. Peaks were annotated for gene promoter using UROPA ^65^and GENCODE version M25 ^66^. Gene promoters were defined as regions within 2kb upstream and 1 kb downstream the gene transcription starting site.

### Loop calling and annotation

Significant loops (q-value<0.05) were called on the downscaled and corrected interaction matrices using FitHIC at 10kb resolution ^67^ and annotated for CTCF peaks and gene TSS using pairToBed ^68^.

### Differential aggregated peak and TAD analyses

Aggregated peak and TAD analysis on downscaled and corrected interaction matrices was performed using GENOVA at 10kb resolution ^41^. For aggregated peaks analyses, the union of loops called by FitHIC in WT and mutant CTCF for each pairwise comparison was used to generate and compare aggregated interaction at loop anchors. For aggregated TAD analyses, the insulation score was first calculated to identify TAD and TAD boundaries. The union of TADs called by GENOVA in WT and mutant CTCF was used to generate and compare aggregated intra and inter-TAD interactions.

### Differential loop analysis

Differential loops (adjusted p-value<0.05) were called for each pairwise comparison between WT and mutant CTCF using MultiHiCCompare which performed a cross-sample normalization before testing for differential loops using an exact test ^69^. Enrichment for differentially expressed genes among differential loops was tested using logistic regression after annotating loop anchors with gene TSS.

## Extended Data

**Extended Data Fig. 7:**
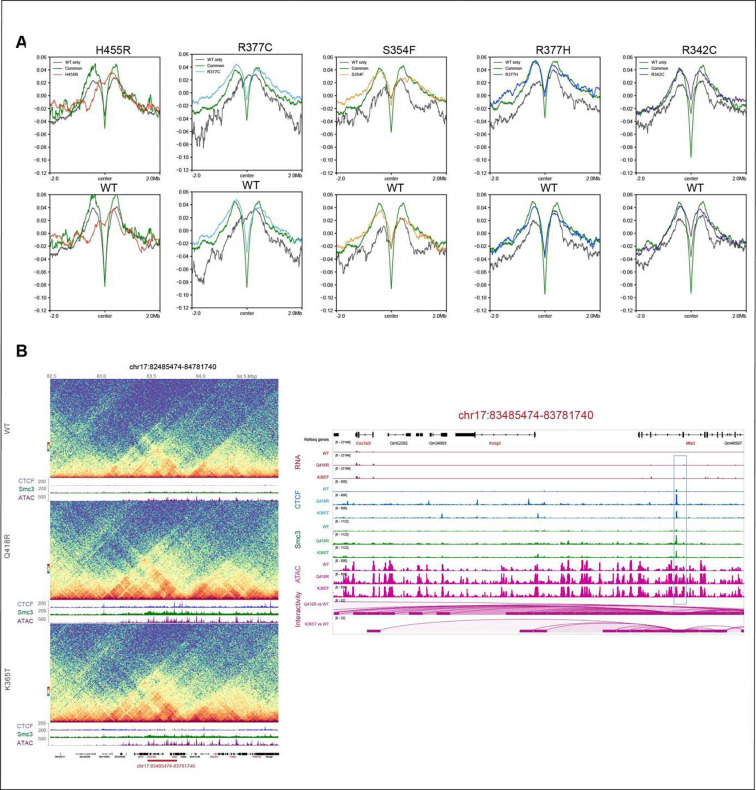
CTCF mutations alter insulation and chromatin interactivity. (**A**) Profiles show the aggregated insulation score at WT only, common and mutant only binding sites for four mutants. The profiles for the remaining mutants are shown in Extended Data Fig. 6F. (**B**) Example of a locus on chromosome 7 (red rectangles) with a direct effect of gain in CTCF binding and chromatin interactivity. The left panel shows the Hi-C interaction matrices in WT, Q418R and K365T with both gain of intra- and inter-TAD interactions in the mutant compared to WT. The left panels show the zoom-in tracks of CTCF (blue) Smc3 (green), ATAC (red) and significant differential chromatin loops (purple) with one anchor overlapping the differential CTCF binding sites. Overexpressed genes are highlighted in red. Of note, only gained interactions were detected at these loci. This analysis was performed at 10 kb resolution.

## Data Availability

All raw and processed sequencing data files are deposited at NCBI’s Gene Expression Omnibus (GEO) and will be available to public on publication of the manuscript.
